# Air-breathing synchrony in juvenile *Arapaima gigas* reveals collective coordination under individual physiological constraints

**DOI:** 10.1038/s42003-026-10472-w

**Published:** 2026-06-17

**Authors:** Palina Bartashevich, Fritz A. Francisco, Alessandra Escurra-Alegre, Fabian Schäfer, Sven Wuertz, Jens Krause, Werner Kloas, David Bierbach

**Affiliations:** 1 Faculty of Life Sciences, Albrecht Daniel Thaer-Institute of Agricultural and Horticultural Sciences, Humboldt-Universität zu Berlin, Berlin, Germany; 2 Cluster of Excellence ‘Science of Intelligence’, Technical University of Berlin, Berlin, Germany; 3 Department of Biology, University of Massachusetts Boston, Boston, MA USA; 4 Department of Fish Biology, Fisheries and Aquaculture, Leibniz-Institute of Freshwater Ecology and Inland Fisheries, Berlin, Germany; 5 Faculty of Life Sciences, Institute of Biology, Humboldt-Universität zu Berlin, Berlin, Germany

**Keywords:** Experimental evolution, Animal behaviour

## Abstract

Animal collectives can exhibit striking synchrony in behavior even when members differ consistently among each other when alone. While synchrony in collective movements or signaling as found in schooling fish or flashing fireflies is well-studied, these behaviors typically do not require individuals to compromise physiological needs when adjusting direction, speed, or timing, as they are generally almost cost-free to produce. In contrast, less is known about how individuals coordinate state-dependent behaviors shaped by internal physiological needs that may limit their ability to conform. We address this question by combining agent-based modeling with empirical observations of a distinctive case of synchronization in fish – the collective air-breathing of juvenile *Arapaima gigas*. We show that individuals differ consistently in surfacing rhythms when alone, yet in a large shoal of about 200 same-aged individuals, a substantial portion of the group surfaces within the same second. Our analysis, supported by individual-based simulations of inherently non-periodic coupled oscillators (units that act stochastically in isolation), reveals a simple social interaction rule by which synchrony emerges despite individual variation in surfacing rhythms. The model, matched to our empirical data, suggests that assortative social responsiveness (“cluster synchrony”) can buffer internal constraints, enabling coordination without overriding individual physiological limitations.

## Abstract (web summary)

Juvenile *Arapaima gigas* - world’s largest freshwater fish - show high degrees of synchrony when air-breathing together despite individual differences. Agent-based models and empirical data suggest assortative social responsiveness as mechanism.

## Introduction

Animal collectives are capable of performing behaviors with a high degree of synchrony, like instant direction changes in shoaling fish, flocking birds, or walking locusts^1–3^, synchronized flashing of fireflies^4^ or vocalization in frogs^5^. Synchrony, e.g., the collective performance of a certain behavior at almost the same time by several group members, is often formally defined as the adjustment of individual activity patterns due to social coupling among partaking group members^6^. In animal groups, synchronous behaviors can emerge when individuals follow simple social interaction rules^7–9^ as well as possess some degree of anticipatory abilities towards the future actions of their neighboring group mates^10,11^. Interestingly, individuals in groups can synchronize behaviors for which they differ consistently among each other when observed in isolation^12,13^. Causes for these consistent individual differences in behavior are manifold and comprise factors like age (and experience), sex, personality, physiology, or morphology^14,15^. However, it is still not entirely understood how these consistent differences in behavior at the individual level are (socially) integrated into synchronized behaviors at the collective level^16–18^, especially when individuals face trade-offs between intrinsic behavioral rhythms and the benefits of group synchrony^19–23^. In such cases, individuals have to compromise between their own rhythms and those of their group members to align with the group.

While synchrony in collective movement or signaling has been widely studied^3,4,20,24–26^, these behaviors typically do not require individuals to constrain their individual physiological needs (e.g., fundamental requirements to maintain homeostasis) when adjusting direction, speed, or timing to align with the group^26^. One fascinating example is found in male fireflies that synchronize their individual light flashing to induce females’ responses^4,27–29^. When alone, fireflies either have intrinsic regular flashing frequencies (i.e., *Pteroptyx malaccae*, see ref. ^30^) or flash more or less irregularly (*Photinus carolinus*)^31–33^. In groups, social coupling   where individuals integrate behaviors of others into their own behaviors— allows them to synchronize towards a certain group flashing frequency that can be highly periodic even when individuals alone would flash irregularly^25,27,32^. However, the synchronization of light flashing does not entail significant physiological compromise for an individual male, as it involves minimal energetic costs^26^. In contrast, to assess the trade-offs and opportunity costs inherent to synchronized behaviors, it is important to focus on systems where individual rhythms are physiologically constrained or exhibit limited flexibility, as these constraints and the level of compromise individuals are able to accept likely play a role in the evolution of grouping behavior.

In this study, we investigated air-breathing behavior in fishes that is performed both in groups and by individuals alone. Several fish species possess both an air-breathing organ as well as functional gills to take up oxygen from the water^34–37^. The control of the air-breathing is driven by oxygen chemoreceptors that monitor water and blood oxygen levels^34,37^, and thus breathing intervals of individuals of the same species are found to differ significantly among each other due to among-individual variation in metabolic oxygen demand (i.e., ref. ^38^). Although these individual differences in the underlying physiological mechanisms and thus physiological needs may constrain an individual’s flexibility to adjust their own breathing rhythms, several fish species exhibit synchronized collective air-breathing where individuals surface together only a few seconds apart, and these “breathing clusters” are interspersed by minute-long non-breathing periods^38–41^. In turn, this synchrony in breathing may come at the cost of shorter breathing intervals in groups compared to when individuals are alone. For example, catfish have been shown to breathe much more frequently in groups than they would do alone, and much more frequently than would be physiologically assumed^38^.

While fish become exposed to omnipresent aerial and terrestrial predators when breaking the water surface to take a breath^39,42^, doing this as a group is assumed to provide risk-reducing advantages^43,44^, especially in bird-fish interactions^45,46^. Although larger groups may cause greater water ripples when breathing together, thus attracting surrounding predators stronger^47^, the benefits of breathing collectively appear to outweigh these costs. In general, the larger the group (I) the lower an individual’s probability of being attacked as the risk is spread across many individuals (dilution effect), (II) the higher the ability to detect a predator (many eyes effect) and (III) the higher the chance the predator might struggle to focus and handle its prey (confusion effect) (see ref. ^43^). Interestingly, it has been proposed that the frequency at which prey animals perform any behavior that exposes them to predators would be an important indicator of the predation regime the prey is facing as well as the prey’s adopted anti-predator strategy^48^. When predators appear at random (such as a hunting raptor that suddenly appears and immediately attacks), regular behavioral patterns should be adopted. In turn, variability (e.g., characterized by geometric or exponential distributions) in behavioral patterns should be favored in the case of stalking predators that might be around for long periods scanning for prey to appear (see refs. ^49–51^).

For our understanding of synchronized collective air-breathing in fish, it is crucial to study how strongly individuals differ among each other in their breathing frequencies and how individuals can compromise their individual oxygen needs in order to take part in the collective breathing events. Thus, investigating possible mechanisms of collective synchrony in air-breathing fishes has the potential to inform other disciplines (e.g., behavioral ecology, neuroscience, robotics, and economics) where a similar coordination of individuals with individual constrains are common scenario.

As a case study, we investigated individual and collective air-breathing of juvenile *Arapaima gigas*. This obligate air-breathing fish is assumed to represent the largest-scaled freshwater bony fish on earth^52,53^ and inhabits wide areas of the Amazon basin^54,55^. In this more than 23 million years old species, adults are reported to exceed 3 m in size and 250 kg in weight^56^. Anecdotal reports hint towards many hundreds of finger-long juveniles performing air-breathing together. In this paper, we present the first empirical descriptions of temporal synchronization in air-breathing within shoals of juvenile *Arapaima gigas*. We hypothesized that the time between successive breaths in our study species should follow an exponential distribution, as such a strategy would imply random appearances at the surface, making it impossible for predators to anticipate the next surfacing event. Since earlier work on air-breathing catfish^38^ imply that fish adapt their air-breathing frequencies in dependence on social context, this also leaves the possibility that juvenile Arapaima may exhibit different breathing rhythms when alone or in a group.

To uncover the underlying mechanisms, we adapted a generic stochastic model based on integrate-and-fire dynamics of non-periodic oscillators^27^ to explain how individuals adjust their inherently different surfacing needs (driven by oxygen demands) to achieve group-level synchrony at the empirically observed breathing frequencies. We first observed single individuals as well as a shoal of ca. 200 same-aged juvenile fish in an indoor aquaculture facility (see “Methods”) and asked the following questions: (1) Do individual juvenile *Arapaima gigas* differ among each other in their spontaneous air-breathing frequency when alone as known from other species? (2) How strongly synchronized are breathing bouts in large shoals on a temporal scale and how many shoal members manage to take part in such synchronized breathing events? (3) Do fish in the shoal breathe more frequently than individuals alone, as known for catfish, and do breathing intervals in single individuals and/or shoals follow an exponential distribution that may hamper predators from predicting future appearances at the surface (*sensu*^48^)? Using agent-based modeling, we studied (4) how the degree of intra- and inter-individual variation in individual air-breathing frequencies may influence the collective breathing performance in terms of synchrony and frequency. As there is no suitable experimental method to selectively change the air-breathing frequencies of certain shoal members yet, we simulated in silico shoals that were either homogenous or heterogeneous in regard to their members’ breathing rhythms (“breathing types”) and compared their collective breathing interval distributions with those observed in the live shoal. By varying proportions of distinct breathing types within in silico groups, we asked (5) which breathing types are most influential for the collective air-breathing behavior, e.g., are there certain “key stone individuals” that may dominate the collective breathing performance. Finally (6), we proposed an interaction mechanism that allows individuals to minimize compromise in breathing frequencies (lowest deviation from individual breathing frequency when alone) while maximizing the number of individuals taking part in the synchronous breathing events, matching our group-level empirical observations.

## Results

### Variability in the individual breathing behavior of juvenile *Arapaima gigas*

**Fig. 1:**
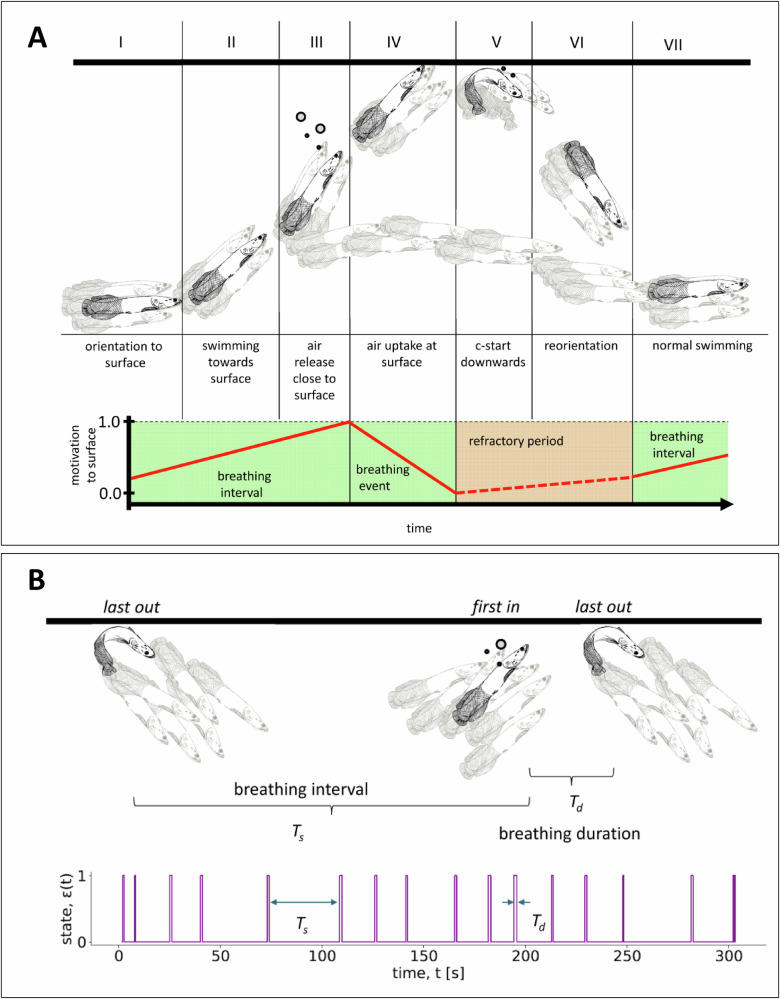
Air-breathing in juvenile Arapaima. **A** The different phases of the breathing sequence (for details, see Table 1) along with the modeled motivation to surface in relation to the real breathing sequence. Note: during the refractory period, fish are unresponsive to social cues (red background), thus their motivation to surface is changing only at their intrinsic rate without social influence from neighboring individuals. **B** Schematic view of the interval scoring for breathing events and the duration of the breathing. For individuals, breathing intervals ${T}_{s}$ were scored as the time between an individual diving down (phase V) and starting to release air (phase III), while breathing duration ${T}_{d}$ is the time between phase III (release of air) and phase V (diving down). For the shoal, breathing intervals ${T}_{s}$ were defined as the time between the last fish diving down to the first fish releasing air again (analogous to phase III and V). In contrast to the isolated individuals, different fish determined the start and end of the breathing event in the shoal. Breathing duration ${T}_{d}$ was scored as the time between the first fish to release air bubbles and the last fish diving down from the surface.

**Fig. 2:**
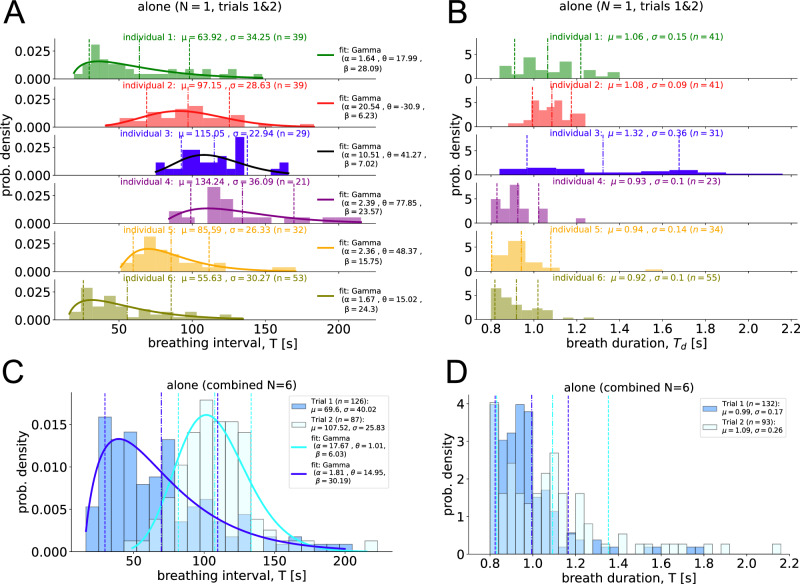
Behavioral variation in the individual breathing behavior of juvenile Arapaima. **A** Breathing intervals and respective **B** breathing durations for each of *n* = 6 individual fish recorded in isolation. Shown is the recorded data for both trials, as well as fitted Gamma distributions (KS-test always not. sig.) with shape parameters $\alpha > 1$ (indicating that the distribution has a peak and is unimodal), location parameters *θ*, and scale parameters *β* (determining the dispersion of the distribution) for each individual fish separately. **C** The combined breathing intervals and **D** breathing durations of all six individual fish recorded in isolation during the first trial (in blue) and the second trial (in cyan). The distribution of the breathing intervals during the first trial and the second trial is fitted with Gamma distributions (trial 1: $\alpha =1.81$, $\theta =14.95$, $\beta =30.19;$ goodness-of-the-fit: KS-statistic = 0.045, *p*-value = 0.95; trial 2: $\alpha =17.67$, $\theta =1.01$, $\beta =6.03;$ goodness-of-the-fit: KS-statistic = 0.074, *p*-value = 0.70).

We recorded the breathing behavior of 6 individuals (body length = 29.8 cm ± 0.8 cm SD). Each individual was observed twice for 1 h for 2 days, with 1 day off between observations. Observations took place in a white, acrylic tank (100 ×100 cm^2^) filled to 15 cm water level. We scored both the time interval between breathing events as well as the length of those events (Fig. 1A). Individuals differed consistently among each other in the intervals between spontaneous air-breathing when alone (sig. Repeatability of 0.53 [0.27–0.77], LRT: *chi²* = 117, *P* < 0.001, Fig. 2A). We further found a significant habituation effect with longer breathing intervals during the second trial (avg. first observation: 69.6 s ± 40.0 SD; avg. second: 107.52 s ± 25.83; sig. effect of trial in LMM: *F*_1,206_ = 99.8, *P* < 0.001; Fig. 2C). Similarly, the duration of the air-breathing behavior was different among individuals (sig. Repeatability of 0.45 [0.21–0.71], LRT: *chi²* = 89, *P* = 0.01, Fig. 2B) and fish took on average longer to breathe in the second trial (avg. first trial: 0.99 s ± 0.17 SD; avg. second trial: 1.09 s ± 0.26; sig. effect of trial in LMM: *F*_1,218_ = 16.9, *P* < 0.001, Fig. 2D). Although we found evidence for the presence of significant individual differences in breathing intervals, we can further classify the individuals as either fast (id 1 and 6), medium (id 2 and 5) or slow (id 3 and 4) breathers (referred to as “breathing types”) based on their average breathing intervals (Fig. 2A). For both trials, air-breathing durations and subsequent intervals were positively correlated (Pearson’s *r*; first observation: *N* = 126, *r* = 0.282, *P* < 0.001, Supplementary Fig. S3A; second observation: *N* = 87, *r* = 0.276, *P* = 0.01, Supplementary Fig. S3B), indicating that on average a longer time since the last breath led to a longer subsequent breathing duration.

### Synchrony in collective breathing behavior of juvenile *Arapaima gigas*

**Fig. 3:**
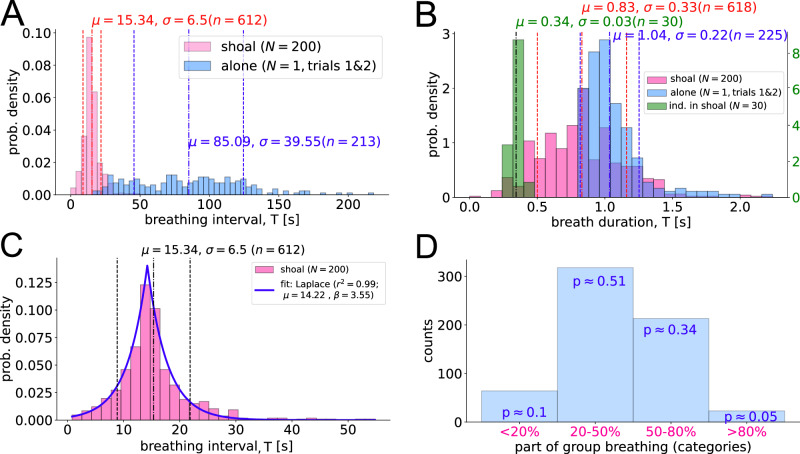
Individual and collective breathing behavior of juvenile *Arapaima gigas*. **A** Histograms of breathing intervals for the shoal (pink) and for individuals measured in isolation (blue). Shoal intervals were defined as the time between successive collective surfacing events (from the first fish surfacing in one coordinated event to the first fish surfacing in the next). Individual breathing intervals were measured from individual fish tested alone (outside the shoal) and pooled across six individuals. **B** Histograms of the breathing duration of the breathing events for the shoal (in pink), for individuals in the shoal (in green), and individuals recorded alone (in blue, both trials combined). **C** Laplace fit for the breathing intervals in the shoal with estimates for the location parameter, the scale parameter, as well as goodness-of-fit. **D** Proportion of the fish participating in the collective breathing events. Shown categories are based on the estimates that range from less than 20% fish partaking to more than 80% partaking (“<20%”, “20–50%”, “50–80%”, “>80%”).

In the observed shoal (ca. 200 juvenile Arapaima; body size ca. 30 cm, same cohort as for individual behavior, see Fig. 1B for a schematic description of the breathing events), we found individual fish to perform very short air-breathing events; three times shorter than the air-breathing duration of the individually observed fish (individual in shoal: 0.34 s ± 0.03 SD, *N* = 30; individual alone: 1.03 s ± 0.22, Fig. 3B). We recorded 618 collective breathing events, and in more than 85% of these events, between 20% and 80% of the entire shoal participated in the collective breathing (Fig. 3D). Individuals were swimming most of the time very close in one shoal in a wall or cube like formation (Supplementary Video 1) and fish closest to the surface were taking part in the air-breathing (Fig. 1A; Supplementary Video 1 and 2). The duration of a collective breathing was on average 0.83 s (±0.33 SD) long (Fig. 3B), and the more fish taking part, the longer the breathing bout (Spearman’s *r*_duration-partaking_ = 0.302, *p* < 0.001, *N* = 618; Supplementary Fig. S4A). This shows that even when about 100 individuals are taking a breath together (=50% of the shoal), this just lasts for about as long as it would take a single individual in isolation to take a breath alone (Fig. 3B). This further exemplifies the extreme degree of synchronization of the collective air-breathing in this species. Intervals between collective breathing events were on average 15.34 s (±6.5 s SD), thus much shorter as found for individuals alone (Fig. 3A). There is also a positive correlation between the proportion of the shoal taking part in a breathing event and the length of the subsequent collective breathing interval (Spearman’s *r*_interval-partaking_ = 0.09, *p* = 0.021, *N* = 612, Supplementary Fig. S4B), indicating that the more fish took part the longer the shoal waits until the next breathing is initiated. The distribution analysis of the individual and collective breathing intervals revealed that individual breathing follows Gamma distributions (Fig. 2A, for details see Methods), while the collective breathing shows strong evidence for a Laplace distribution (i.e., double-exponential distribution, Fig. 3C, *r*² = 0.99; for details see “Methods”).

### Agent-based simulations of air-breathing (surfacing) coordination in groups

**Fig. 4:**
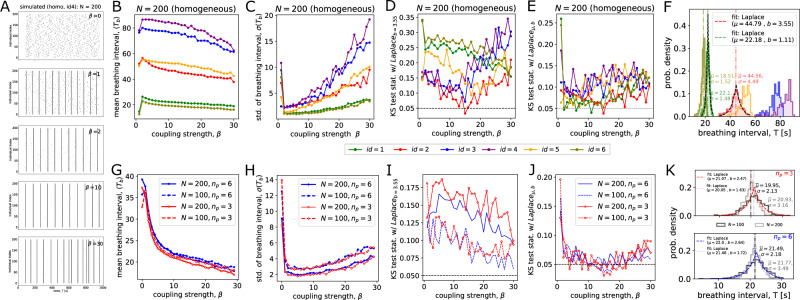
Effect of individual breathing diversity on the collective air-breathing in simulated homogeneous and heterogeneous groups. **A** Example of simulated breathing intervals for a group (*N* = 200) composed of only id=4 breathing type at different social coupling strengths *β*. At $\beta =1$, synchronous collective events occur, but many individual unsynchronized breathing events remain between them (visible as isolated dots between the vertical alignments), indicating only partial synchronization; at larger *β* values, these sporadic in-between events disappear, reflecting full collective synchronization. **B** Simulated mean breathing intervals and **C** their standard deviation for homogenous groups composed of either one of the 6 individual breathing types. **D** Two-sided Kolmogorov-Smirnov test results verify at each β whether breathing intervals in the modeled groups come from the Laplace distribution with the empirical scale parameter $b=3.55$ or **E** from any Laplace-like distribution without fixed parameters. The KS-test values above 0.05 indicate rejection of the null hypothesis, meaning the collective breathing intervals of the simulated group do not follow a Laplace distribution. **F** Depiction of the model-generated collective breathing interval distributions for six homogenous independent groups with β parameters corresponding to their best fits to the Laplace distribution ($\beta =11$ for ${id}\in ({\mathrm{1,3,4,6}})$ and $\beta =15$ for ${id}\in ({\mathrm{2,5}})$). **G** Simulated mean breathing intervals and **H** their standard deviation for heterogeneous groups composed of either only ${n}_{p}=3$ or all ${n}_{p}=6$ different individual breathing types for group sizes of $N=100$ and $N=200$. **I** KS test results verify at each β whether breathing intervals in the modeled groups come from the Laplace distribution with the empirical scale parameter $b=3.55$ or **J** from any Laplace-like distribution without fixed parameters. **K** Depiction of the model-generated collective breathing interval distributions for heterogeneous independent groups of *n*_*p*_ =  3 and *n*_*p*_ = 6 with $\beta =14$ for group size of $N=100$; as well as for group size of $N=200$ (here with $\beta =15$ in case of *n*_*p*_ = 3 and *β* = 13 in case of ${n}_{p}=6$). All distributions were generated from 10 simulations, 1000 time steps each, with ${dt}=0.01$ and the refractory period *τ*_ref_ = 1.1.

First, we considered a group of *N* = 200 (matching the shoal size of the empirical data) agents where all individuals shared the same breathing type (homogeneous group). Here, we sampled for each agent *i* individual breathing intervals ${T}_{{si}}$ from one of the Gamma distributions derived from the experimental data of isolated individuals (Fig. 2A), corresponding to the given breathing type. Depending on the breathing type, individuals exhibited different breathing interval characteristics within the group (Fig. 4B–E). As the strength of social interactions (coupling strength *β*) increased from 0 to 1, individuals started to adjust their breathing, resulting in an initial increase in breathing intervals (Fig. 4B) and a sharp decrease in their standard deviation (Fig. 4C), while the group remains only partially synchronized (Fig. 4A and Supplementary Fig. S5A). With further increase of coupling strength $\beta > 1$, as the group approaches full synchrony (Fig. 4A and Supplementary Fig. S5A), this trend was reversed as the mean breathing interval decreased (Fig. 4B) and its standard deviation increased (Fig. 4C), regardless of the breathing type. This means that with a strong coupling strength among individuals (*β* > 10), the synchronous collective breathing pattern became more irregular rather than periodic, i.e., it did not follow a fixed interval (e.g., see Fig. 4A and Supplementary Fig. S5A–C).

In the next step, we compared the breathing interval distributions generated by the model for different coupling strength *β* with the experimentally obtained data for the shoal (see “Methods” for details). While we found some breathing types in a homogeneous group to follow Laplace-like distributions in their collective breathing intervals, either the location parameter (breathing type id = 2: $\mu =44.79$, Fig. 4D, F) or the scale parameter (breathing type id = 1: $b=1.11$, Fig. 4E, F) did not match with empirical data (see Supplementary Note 1). Notably, the Laplace-like distribution of breathing intervals was observed only for strong social coupling between individuals ($\beta =11$ for breathing type id = 1 and $\beta =15$ for breathing type id = 2).

Since simulations of groups with homogeneous breathing types did not match the empirical collective breathing interval distribution, we simulated heterogeneous group compositions where all six given breathing types were included. We also tested whether the three main breathing types (e.g., id = 6 as fast; id = 5 as medium; id = 4 as slow; for details see Supplementary Note 2: semi-heterogeneous case), instead of all six, can be sufficient to represent the empirically found collective breathing intervals. By studying the statistical properties of simulated groups of *N* = 100 and *N* = 200 individuals—each composed of either all six or only three breathing types, with each type in equal proportion relative to a group size—our simulations showed that as social coupling strength increased the mean breathing interval in heterogeneous groups declined more rapidly compared to the homogeneous groups (Fig. 4G vs. Fig. 4B). This can be explained by a convergence of the breathing interval to the fastest breathing type present within the group as exemplified by our analysis of semi-heterogeneous groups (i.e., groups containing members with just two distinct breathing types; see Supplementary Note 2: semi-heterogeneous case). The standard deviation of the breathing interval in the group with diverse (i.e., more than 2) breathing types tended to increase slower with increasing coupling strength compared to the homogenous groups (Fig. 4H vs. Fig. 4C), while it, in general, followed the pattern of the fastest breathing type in the group (i.e., id = 6 in Fig. 4C; also see Supplementary Note 2: semi-heterogeneous case).

We found collective breathing dynamics differ between group sizes but not between six- and three-breathing type compositions (Fig. 4G–J), most probably because within the underlying 6 individual breathing types, there were already 3 distinct main breathing types with 2 individuals each (see also semi-heterogeneous cases in Supplementary Note 2). Consistent with ref. ^27^, the mean and the standard deviation of the group breathing intervals increased with decreasing group size (Fig. 4G, H).

All considered heterogeneous group compositions generated Laplace-like distributions of collective breathing intervals (Fig. 4J). However, none of them matched the distribution of breathing intervals with empirical scale parameter *b*, regardless of the coupling strength *β* (Fig. 4I), and characterized by a narrower distribution shape overall (Fig. 4K). This indicates that the included breathing types may not represent the full range of breathing types present in the observed Arapaima shoal. Notably, our selected breathing types for the three-type groups did not follow any Laplace-like distribution in a homogenous case (i.e., id ∈ (4, 5, 6), also see Fig. 4E) but did so when analyzed as a heterogeneous group (Fig. 4J). Despite this, when interacting together, the breathing dynamics of such three-breathing types compositions resembled those of the six-personality composition (${n}_{p}=3$ vs. ${n}_{p}=6$ in Fig. 4K), which includes breathing types that can exhibit a Laplace-like distribution in the homogenous case on their own (i.e., due to the intra-individual variation). This suggests that the Laplace-like distributions of the distinct behavior in focus—collective synchronous air-breathing—are an indication of a heterogeneous group member composition in regard to the individually performed behavior.

### Impact of varying breathing type ratios on the collective air-breathing in heterogeneous groups

Above, we demonstrated how individual differences—both within and between individuals—affect collective air-breathing behavior, assuming equal proportions of each breathing type within the group. Here, we explored how varying these proportions will affect the collective air-breathing dynamics by adjusting the extremes —decreasing the number of “slow breathers” and increasing the number of “fast breathers”. We focused on the three-breathing type group compositions, sampling individual breathing intervals from the fitted Gamma distributions of individuals 4, 5, and 6 when tested alone (see Fig. 2A). We maintained the proportion of “medium” (id = 5) breathers at ⅓ of the group size *N* and varied the number of “fast” (id = 6) breathers ${N}_{{fast}}$ from *0* to ⅔. The number of “slow” (id = 4) breathers is respectively adjusted to be the remaining fraction (⅔*$N-{N}_{{fast}}$).

**Fig. 5:**
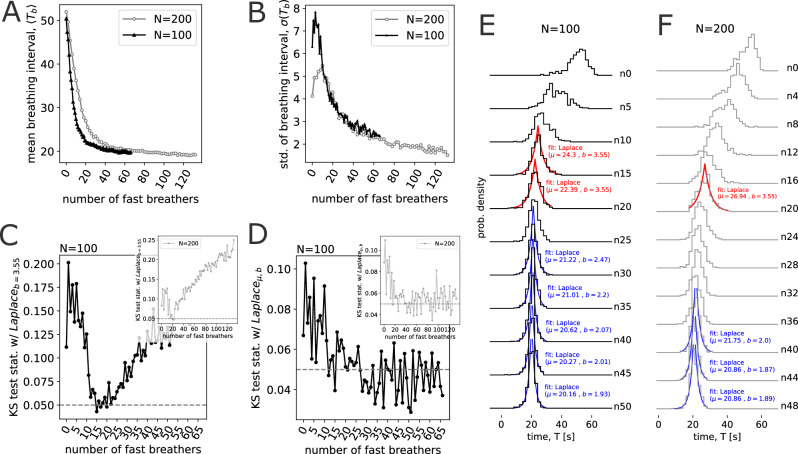
Impact of varying ratios of breathing types on the collective air-breathing in simulated heterogeneous groups. **A** Simulated individual breathing intervals (means) and **B** their standard deviations of the mean in a heterogeneous three-breathing-type group composition as a function of the number of “fast” breathers ${N}_{{fast}}$, for different group sizes *N*. The number of present individuals of each of the types is recalculated as (${N}_{{fast}}$, ⅓*N, ⅔*$N-{N}_{{fast}}$) for “fast”, “medium”, and “slow” breathing types, respectively. **C** Matching of simulated distributions of collective air-breathing intervals to the empirically found Laplace distribution with the scale parameter $b=3.55$ or **D** to any Laplace-like distribution without a fixed scale parameter. Histograms of the simulated breathing interval distributions for groups of *N* = 100 (**E**) and *N* = 200 (**F**) individuals, with increasing numbers of “fast” breathers (e.g., n0 corresponds to their absence). In (**A**–**F**), the strength of the social coupling is set to $\beta =14$. All distributions were generated from 10 simulations, 1000 time steps each, with ${dt}=0.01$ and the refractory period *τ*_ref_ = 1.1.

The mean breathing interval of individuals in a group decreased asymptomatically as the number of fast breathers was increased (Fig. 5A). The standard deviation of the breathing intervals initially increased with the introduction of fast breathers, peaking at 10 individuals in the case of *N* = 200 and at 3 individuals for *N* = 100. As more fast breathers were added, the standard deviation started to decrease, indicating that the group breathing became more regular with the addition of fast breathers (Fig. 5B). Once there are 10 fast breathers in a group of either *N* = 100 or *N* = 200 individuals, their influence on the variability of the breathing intervals became comparable between the considered group sizes (Fig. 5B).

We found that when proportions of fast breathers started to exceed about 10% in a group of $N=200$ and 15–20% in a group of $N=100$, the collective breathing intervals matched Laplace-like distributions (Fig. 5D–F). However, distributions with the empirical scale parameter $b=3.55$ were only found for groups with 10%-15% fast breathers (Fig. 5C, E, F). Notably, when there are no “fast” breathers in a group and “slow” breathers are in the majority (i.e., ⅔ of N), the group with “medium” breathers (that are now the fastest breathers in the group) did not follow a Laplace-like distribution of breathing intervals (Fig. 5D). This means, that a small minority of “fast” breathers—around 10% to 15%, depending on group size—not only replicated the shape of the empirical Laplace distribution for breathing intervals but was also sufficient to significantly accelerate the collective breathing rate, bringing it closer to the empirical location parameter (i.e., $\mu =14.22$; Fig. 3C). However, the remaining deviation from the empirical mean (i.e., $\mu =26.94$ in the case of ${N}_{{fast}}=20$, $N=200$; Fig. 5F) implies that the “fast” breathing type we consider in our simulations might not represent the absolute “fastest” individual type present in the empirical shoal (see Supplementary Note 3.5 and Supplementary Fig. S9).

### Individual compromise and collective partaking rates

While our simulations showed how the empirical Laplace distribution of the collective breathing intervals can emerge in a shoal with heterogeneous individual breathing types, this scenario assumes that all individuals participate in the breathing events (100% partaking). In contrast, our observations of the live fish showed that typically only a fraction of the individuals and not the entire group came to the surface (partial partaking) (Fig. 3D, Supplementary Video 1 and 2). This fraction—in the majority of cases between 20% to 80% of the group—form a synchronized subgroup (cluster), while the fraction of those that do not participate in the surfacing can be seen as a separate subgroup. This behavioral pattern can be referred to as *cluster synchronization*, where the group splits into distinct clusters that exhibit internal synchrony but are not synchronized with other clusters (e.g., refs. ^57–59^). To simulate this, we assume a fully connected network where individuals of each breathing type (fast, medium, slow) form a breathing cluster and the strength of interactions is weaker between individuals of different breathing clusters (inter-cluster coupling, ${\beta }_{{{inter}}_{{clu}}}$) and significantly stronger within the same cluster (intra-cluster coupling, ${\beta }_{{{intra}}_{{clu}}}$; such that ${\beta }_{{{intra}}_{{clu}}}\gg {\beta }_{{{inter}}_{{clu}}}$).

**Fig. 6:**
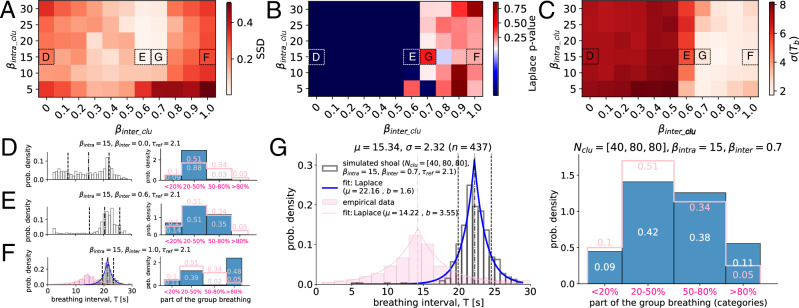
Influence of intra- and inter-cluster coupling strength on the proportion of part-taking individuals and breathing interval distribution in simulated heterogeneous groups. **A** Difference between empirical and simulation-generated probabilities of the proportions of individuals participating in breathing events depicted as the sum of squared differences (SSD) for varying intra- and inter-cluster coupling strengths. **B** Likelihood of encountering a Laplace-like distribution for different combinations of the intra- and inter-cluster coupling strengths based on the KS test statistics. If the *p*-value is below 0.05 (in blue), the intervals do not follow a Laplace-like distribution. **C** The standard deviation of the simulation-generated collective breathing intervals for varying intra- and inter-cluster coupling strengths, indicating the degree of irregularity in the breathing pattern. **D**–**G** Histograms of the distributions of collective breathing intervals and proportions of the group participating in the breathing event in simulation (in blue) versus empirical data (in pink) for four different sets of parameters, highlighted by different corresponding letters in (**A**–**C**). All distributions were generated from 10 simulations, 1000 time steps each with ${dt}=0.01$ and the refractory period ${\tau }_{{ref}}=2.1.$ Simulated groups were composed of $N=200$ individuals from three breathing types with ${ids}\in \left({\mathrm{4,5,6}}\right)$ and different intra- and inter-cluster coupling strengths. Based on the results of the previous section, we assumed fast breathers are in the minority and represent the group with an unequal ratio of fast:medium:slow breathers as 1:2:2, corresponding to breathing cluster sizes of ${N}_{{clu}}\in ({\mathrm{40,80,80}})$ agents.

We identified a transition region at ${\beta }_{{{inter}}_{{clu}}}=0.7$ where the collective breathing behavior dramatically changes. Below the transition (${\beta }_{{{inter}}_{{clu}}}\ll 0.7$), individuals primarily breathe together with individuals of their own breathing types, making the “20–50%” partaking category the most dominant (i.e., with 0.88 probability of occurrence, see Fig. 6D). Since collective breathing intervals are measured from the last individual to dive in one cluster to the first one that is resurfacing (from any cluster), this leads to short but highly inconsistent intervals between breathing events, and thus high standard deviation of the breathing intervals (“D” in Fig. 6C). As a result, the collective breathing intervals failed to form a Laplace-like distribution, regardless of the coupling strength ${\beta }_{{{intra}}_{{clu}}}$ (Fig. 6B). Above the transition (${\beta }_{{{inter}}_{{clu}}}\gg 0.7$), our simulations showed a strong tendency for full synchrony of all individuals in the group during the breathing events (indicated by the increasing dominance of “>80%” partaking category; Fig. 6F), especially when the strength of the inter-cluster coupling exceeded ${\beta }_{{{inter}}_{{clu}}}=1.0$. The standard deviation of the breathing intervals and the shape of their Laplace-like distributions did not change with increasing inter-cluster coupling strength after the transition (Fig. 6F, G). Most notably, our empirical data is best matched around the transition region. Here, the lowest SSD values are found, which indicates the emergence of cluster synchronization with proportions of partaking individuals comparable to the empirical estimations (“E” and “G” in Fig. 6A). Also, Laplace-like distributions of breathing intervals emerge above the transition region (i.e., for ${\beta }_{{{inter}}_{{clu}}} > 0.6$ in Fig. 6B). In summary, at weak inter-cluster and strong intra-cluster coupling (i.e., ${\beta }_{{{inter}}_{{clu}}}=0.7,{\beta }_{{{intra}}_{{clu}}}=15$), our model showed empirical-like shoal partaking behavior in the breathing events with a Laplace-like distribution of collective breathing intervals. Nevertheless, regardless of whether the group exhibited full or clustered partaking, the simulated distributions of collective air-breathing intervals consistently differ from the empirical data, showing either narrower or shifted Laplace-like distributions compared to the empirical fit (Fig. 6G). This suggests that the diversity in the shoal may be higher than three breathing types inferred from isolated experiments, which were used in the simulations (see also Supplementary Note 3.5 and Supplementary Fig. S9).

**Fig. 7:**
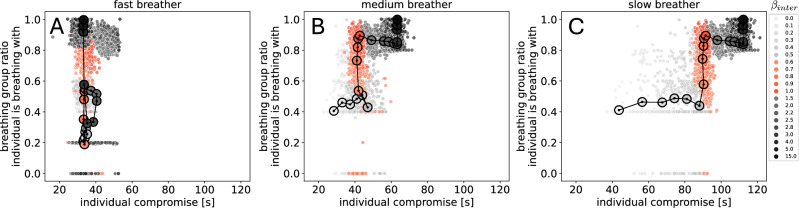
Partaking in simulated collective breathing as a function of individual compromise. Individual compromise (the deviation of an individual’s breathing interval in a group from its mean breathing interval when alone) is plotted against the number of individuals an individual is breathing synchronously with (given as ratio of the whole group). This relationship is analyzed under different inter-cluster coupling strengths ${\beta }_{{{inter}}_{{clu}}}$, while keeping high intra-coupling strength ${\beta }_{{{intra}}_{{clu}}}=15$. In a simulated group of *N* = 200 individuals with a 20%-40%-40% composition of fast, medium, and slow breathers (see Fig. 5), we tracked one randomly selected fast (**A**), one medium (**B**), and one slow (**C**) breather. At ${\beta }_{{{inter}}_{{clu}}}=0$, individuals of each breathing type are only responsive to others of the same breathing type (independent homogeneous groups), thus partaking ratios mirror the fraction of individuals of this breathing type in the shoal (0.2-0.4-0.4) with some breathing events that coincide with other breathing types by chance. Above ${\beta }_{{{inter}}_{{clu}}}=5$ individuals of all breathing types breathe together (full partaking, i.e., non-clustered heterogeneous group). The parameter regime ${\beta }_{{{inter}}_{{clu}}}\in \left[{\mathrm{0.6,1.0}}\right]$ (highlighted in shades of red) marks a transition region in which individuals can increase their participation in collective breathing events without increasing their deviation further from their preferred individual mean breathing interval when breathing alone (i.e., without additional individual compromise). Simulations that best matched the empirical data in terms of partaking and collective breathing interval distribution fall within this regime (${\beta }_{{{inter}}_{{clu}}}=0.7$). Each small dot represents a single breathing event from the simulations with respective inter-cluster coupling strength ${\beta }_{{{inter}}_{{clu}}}$. Big circles indicate mean values across all events and simulations for the corresponding ${\beta }_{{{inter}}_{{clu}}}$, with the line connecting these averages in order of increasing ${\beta }_{{{inter}}_{{clu}}}$. The results in (**A**–**C**) are based on 10 simulations, each running for 1000 time steps with ${dt}=0.01$ and the refractory period *τ*_ref_ = 2.1.

When looking at an individual’s compromise (the deviation of an individual’s breathing interval in a group from the mean breathing interval of this individual when alone) in relation to the proportion of others that synchronize their air-breathing with this individual, our simulations show that slow breathing types can adjust the partaking rate by changing inter-cluster responsiveness ${\beta }_{{{inter}}_{{clu}}}$ (Fig. 7C). This allows individuals to shift from breathing synchronously almost exclusively with others of their own breathing type at inter-cluster responsiveness ${\beta }_{{{inter}}_{{clu}}}=0$ to breathing in full synchrony with all shoal members regardless of breathing types at high inter-cluster responsiveness values (Fig. 7A–C). At inter-cluster responsiveness around the aforementioned transition region (${\beta }_{{{inter}}_{{clu}}}\in \left[{\mathrm{0.6,1}}\right]$), every breathing type in the group can adjust partaking rates without additional costs/compromise (see a sharp jump highlighted in shades of red color in Fig. 7B, C). This means that in this region, even minute changes in responsiveness lead to immense changes in partaking rates while individual compromise is maintained at a similar level for all simulated breathing types. Not surprisingly, though, the range of possible compromise was the smallest for the fast-breathing type (Fig. 7A) and the largest for the slow one (Fig. 7C). This can be explained by the fact that fast breathers cannot extend their breathing intervals as much as slow breathers, who could easily breathe at faster intervals. For fast breathers, the inter-cluster responsiveness only determined the partaking rate, while for slow and medium breathing types, inter-cluster responsiveness also affected the individual compromise. Furthermore, above a certain inter-cluster responsiveness $({\beta }_{{{inter}}_{{clu}}} > 0.8)$, slow and medium breathers have not been found to breathe alone, while this could still happen for fast breathers (for details see SI, Table S1).

## Discussion

Our study provides evidence for the synchronous collective air-breathing behavior of juvenile *Arapaima gigas*, which has not yet been scientifically documented. In a shoal of about 200 individuals kept in an aquaculture facility, we often found more than 100 individuals surfacing together within a second, in several cases, also all 200 individuals. The shoal repeated this highly synchronized behavior almost every 15 s while individuals observed alone showed consistent differences in the frequency of the air-breathing behavior that ranged from about 1 min to more than 2 min. Interestingly, when comparing the distribution of the breathing intervals between isolated individuals and the shoal, we found individual breathing intervals to follow broad gamma distributions, while as a collective, the intervals were Laplace distributed and thus more regular than for individuals alone. Our simulation results illustrated that a *heterogeneous* (in terms of individual breathing intervals) shoal composition is necessary to match the Laplace-like experimentally observed distribution of breathing intervals in a group of *N* = 200 fish. Especially the “fast” breathers seem to be important drivers of the observed interval distributions, with 10% of fast-breathing individuals being enough to significantly accelerate the breathing rhythm of the whole group of 200 to the interval of fast breathers. Using our model, we provide further evidence that the participation rate between 50% and 80% of the shoal members is likely due to a tendency of individuals to breathe within subgroups of similar breathing intervals due to self-sorting and spatial constraints in the shoal—a phenomenon called cluster synchronicity^58,59^. As the surfacing exposes juvenile Arapaima to aerial predators, individuals when alone may breathe less often and at less predictable intervals, while the more regular Laplace distributed breathing in the shoal might be caused by compromises needed to allow individuals with different individual breathing needs to still breathe together as a group—a known anti-predator strategy as well.

Juvenile Arapaima in our study differed vastly in their individual breathing intervals when observed alone, which points towards among-individual variation in metabolic oxygen demands, similar to what is known from catfish^38^. Nevertheless, individual breathing intervals had a broad range, which seemingly allowed the fish to synchronize their air-breathing when together in a shoal, with often about 50% and more of the shoal members breathing at the same time. This temporal synchrony is striking, as other fish species do not show such high temporal coordination in air-breathing^38,40,42^. Perhaps this is due to the fact that other reports stem from non-shoaling or facultative shoaling species, while Arapaima juveniles have been found to strongly shoal and even school, and thus are close together when the air-breathing is initiated. As found in catfish^38^, individuals in the shoal breathe much more frequently than they would do alone, leading us to assume a strong social coupling and making air-breathing in Arapaima—similar to that proposed in catfish—an emergent collective behavior^19^ and an example of social facilitation of behavior^60^.

Distributions of air-breathing intervals of single individuals followed a broad gamma distribution, while those exhibited in the shoal matched a broadened Laplace distribution. The intervals of a behavior that exposes prey to predators are an important indicator of both the predator’s anticipated encounter rates as well as the prey’s anti-predator strategy^49^. For juvenile Arapaima, visiting the surface exposes them to aerial predators that might always be around in their natural habitats, looking for prey to appear. Thus, the observed surfacing at low frequency and at irregular and unpredictable intervals seems to be an adaptive anti-predator strategy for single individuals. For the shoal, however, the surfacing pattern is different, and intervals between subsequent breathing events are more regular. Compared to exponential distributions like gamma, a Laplace distribution has a sharp peak^61^ indicating that the group tends to breathe most frequently at a certain interval, which makes their surfacing behavior potentially more predictable to predators. However, in contrast to fireflies, where their collective flashing might serve as a signal and is thus believed to be most effective if regularly and periodically^62,63^, the collective execution of the air-breathing has most likely an anti-predator function, and we thus hypothesize that the anti-predator benefits of performing the surfacing as a large group outweigh the costs of a higher and more regular breathing frequency. A surfacing shoal might be well visible for any predator that is present anyway^46^—regardless of the frequency range and regularity of the breathing intervals, but predators may be impeded due to confusion or deterrence effects that come along with collectively performed behaviors (see refs. ^64–66^).

We adapted the stochastic synchronization computational framework of^27^ which is based on the integrate-and-fire model of random oscillators. This allowed us to explore possible mechanisms of how Arapaima shoals are able to synchronize the majority of individuals that themselves differ vastly in breathing intervals to perform the air-breathing collectively and almost simultaneously. Our exploration of the model revealed that introducing a refractory period after the breathing event is required to generate a high degree of synchronization in both homogenous and heterogeneous collectives of stochastic agents. This matches with the observation of the live shoal where individuals seem to be unresponsive to social cues of their neighbors once their old air is expelled and until they turn back into the deeper water with a c-start (see Supplementary Video 2). Here, individual fish tracking in 3D^67^ might help to pinpoint the exact time frame during which neighboring cues are ignored, and thus future studies that apply such or similar techniques can also help to add spatial information of the individuals in the shoal to our understanding.

Our analysis further revealed that to match a Laplace-like distribution of the collective breathing intervals, the shoal must be composed of individuals with among-individual differences in their own intrinsic breathing intervals, as exemplified in simulated shoals with either heterogeneous or homogenous member compositions. The resulting peak of the breathing interval distribution in a heterogeneous group aligned with the most frequent interval of the fastest breathing type. This means that we observe a convergence of the collective breathing frequency to the intrinsic frequencies of the fastest breathers, as those fast-breathing individuals have the shortest intervals between subsequent breathings and thus possess the narrowest window for a compromise in the breathing intervals. Fast breathers simply cannot stay longer underwater without breathing, and the social coupling of their conspecifics results in many individuals following them to the surface. Those fast breathers may therefore be seen as key stone individuals^68^ with the strongest influence on the groups’ performances, while our simulations also show that their proportion in the group must be relatively small at about 10%. Increasing the proportion of fast breathers above 10% narrows the collective breathing distribution, reducing the “emergent variability” and making the group’s breathing pattern potentially easier to predict for predators. Our results are consistent with the previous work on leadership in groups (e.g., ref. ^69^), where it was shown that a very small proportion of informed individuals (~10%) is needed to guide the group decisions. Our numerical simulations also suggest that the real fastest-breathing individuals have not been included in our subset of individually tested fish, where we randomly picked 6 individuals from the shoal, and from which we use the data to approximate breathing types in the simulations. While the model successfully replicated the Laplace-like shape of the empirical distribution, the location of the peak remained shifted compared to the empirical data ($\mu =22.16s$ vs. ${\mu }_{{emp}}=14.22s$). Overall, our simulations suggest that shoals can only harbor a certain fraction of fast breathers in order to still possess a somewhat unpredictable surfacing frequency, and thus, balancing selection on intermediate breathing types may come into play.

We observed that not all individuals took part in the collective air-breathing, although sometimes more than 80% or even almost all of the about 200 shoal members surfaced together. As a potential mechanism for this partial participation, we propose that individuals spatially self-assort based on their individual breathing needs (in our simulations, implemented as fast, medium, and slow breathing types). The non-random spatial clustering of individuals with similar behavioral traits has been previously studied in both animal and human groups^70–72^. Arapaima shoals are often layered, resembling a moving cube or wall (“wall-like swimming mode”, see Supplementary Video 1) where the upper layers are closest to the surface and—based on our qualitative observations—surface together while the bottom layers do not participate in the current collective breathing event. Such a self-organization might create spatial constraints, as social coupling in fish is often mediated through visual inputs^8,9^, and with the fast nature of the collective breathing intervals, not all individuals in a large group can see their surfacing shoal mates in time to respond by surfacing themselves. As a result, we could assume stronger social coupling within the same breathing type (a cluster) and weaker social coupling between individuals that are spatially distinct and possibly possess a different breathing type. The implementation of this cluster interaction mechanism allowed us to reproduce part-taking distributions similar to the experimentally observed ones during the collective air-breathing. As the breathing intervals continue to follow Laplace-like distributions, it can be assumed that this interaction mechanism modifies network structure dynamics while preserving the group-level patterns of behavior. Most interestingly, we found a clear transition region at relatively low inter-cluster coupling strength (${\beta }_{{{inter}}_{{clu}}}\in \left[{\mathrm{0.6,1.0}}\right]$) where every breathing type in the shoal can adjust partaking rates at almost the same compromise level. This means that in this region, even minute changes in responsiveness lead to immense changes in partaking rates while individual compromise is maintained at a similar level for all simulated breathing types. This aligns with principles of criticality, which propose that collective systems operate near critical points parameter regimes where small changes in one variable can lead to significant changes in system-wide behavior^73–75^. In this regard, our model shows that by tuning the intra-cluster responsiveness between individuals of different behavioral types, a heterogeneous group can self-adjust to balance the inherent trade-offs between minimizing individual compromise and maximizing collective partaking rate. We believe that such a mechanism can offer insights not only into biological coordination but also into the design of decentralized control in robotic swarms under diverse individual constraints.

We acknowledge the limited sample size of our empirical part of the study. However, as *Arapaima* in South America or Southeast Asia are bred in highly turbid outdoor facilities with little to no possibilities to observe their behaviors underwater, our continuous four-hour recordings of a shoal represent a rare data set and thus a fruitful starting point for the investigation of this 23 Mio years old species. Based on the species’ old phylogenetic age, it is exemplified that collective behavior must have evolved early on, and similar assumptions have been drawn from fossils of highly polarized fish^76^. In future work, spatially explicit modeling combined with recording of the 3D positions of the individual fish in the shoal would provide experimental data on the social network structure and their dynamics during the coordinated air-breathing. Also, more systematic variation of live shoal compositions and shoal sizes would allow further verification of the predictions derived from our simulations.

## Methods

### Study organisms and sampling

We investigated juvenile *Arapaima gigas* (age about 3 months) that were imported from Peru to Germany as fingerlings and raised under indoor Recirculating Aquaculture Systems (RAS) conditions at the Manich Food Innovations GmbH (MFI) in Dingelstedt, Thuringia, for one month prior to our observations. Fish were fed with *Chironomid* larvae multiple times a day and kept at 28 °C water temperature under 12:12 light:dark cycle throughout. We aimed at observing breathing behavior both in isolated juveniles (individual breathing) as well as in the whole shoal of about 200 individuals (collective breathing). We cannot provide the exact number of individuals in the shoal because counts were conducted weekly by the local animal caretakers. However, due to unreported deaths and potential cannibalism, we decided to omit exact numbers here. Further, the number of individuals housed together as well as the maintenance conditions were chosen by MFI to ensure optimal growth and health conditions. Our non-invasive observations have been approved both by MFI and IGB internal animal welfare boards, and since all video recording took place during regular maintenance or health assessment work, there is no further approval by other authorities needed. During our recordings, we have complied with all relevant ethical regulations for animal use.

In general, there are seven sequential phases of the juvenile breathing behavior that can be distinguished (Fig. 1A; Table 1, see also ref. ^77^ for a more complete description of Arapaima behaviors, see also Supplementary Videos 1, 2).

**Table 1:** Description of juvenile Arapaima air-breathing behavior

Phase 1 (orientation to surface)	The whole shoal swims in the middle of the water column and, at some point, orients towards the surface.
Phase 2 (swimming towards the surface):	All fish or only fish in the upper part of the shoal swim straight or with pointed angle towards the surface. Individual fish follow each other to the surface until the first fish return form surface; when fish return into the shoal, others rarely start to move to the surface, and surfacing attempts can be aborted if returning individuals are encountered.
Phase 3 (air release close to the surface)	When approximately ½ to 1 body length away from the surface, individuals release air through gill slits, well visible as bubbles. This can be considered the point of no return, and we never observed any aborted surfacing attempts after an individual released air.
Phase 4 (air uptake at the surface):	Individuals reach the surface and quickly swallow fresh air.
Phase 5 (c-start downwards):	Immediately after the fresh air is swallowed, individuals bend with a c-start like movement downwards and swim away from the surface, often with small bubbles released out of gill slits.
Phase 6 (reorientation):	Individuals orient towards others and integrate into the shoal.
Phase 7 (normal swimming)	The shoal swims as a whole in the middle of the water column again.

We describe the air-breathing behavior as a sequence of seven distinct phases. Please see Fig. 1A for a schematic depiction.

### Individual breathing

We recorded the breathing behavior of 6 individuals (body length = 29.8 cm ± 0.8 cm SD) that were separated in quarantine tanks for routine check-ups at IGB Berlin. Each individual was observed twice for 1 h for 2 days, with 1 day off between observations. Observations took place in a white, acrylic tank (100 × 100 cm^2^) filled to 15 cm water level with aged tap water at 28 °C. We recorded the fish with a Basler acA2040-90um camera with 2048 × 2048 pixel and 25 fps and scored the time (in frames, converted to seconds) between breathing events. We manually scored the breathing duration ${T}_{d}$ as the time between phase III (air release close to the surface) and phase V (c-start downwards) (see Fig. 1AB). Breathing intervals ${T}_{s}$ (Supplementary Fig. S1) were computed as the time between an individual diving down (phase V) and starting to release the air bubbles (phase III, Fig. 1AB).

### Collective breathing

The shoal (ca. 200 juvenile Arapaima; body size ca. 30 cm, same cohort as for individual behavior) was recorded through a glass window in their rearing tank wall (3 × 1 × 1 m) with a Basler acA2040-90um, 2048 × 2048 px camera and a custom recording software^78^ at a frame rate of 25 fps (later converted to 30 fps for technical reasons). We recorded the shoal for 219 min in one day to avoid any major disturbances of the fish through multiple recording sessions (Supplementary Fig. S2). This is important as these fish often stop feeding when subject to prolonged handling (per. observation). From the videos, we scored manually the duration of and the interval between collective breathing events to the nearest frame (Fig. 1B). We defined a collective breathing duration as the time between the first fish releasing air bubbles on its way to the surface until the last fish was diving down from the surface. This is analogous to phase III and V for individual breathing, however, in the shoal, different fish might have determined the start and end of the breathing event (first fish in - last fish out approach, see Fig. 1B). Breathing intervals for the shoal were defined as the time between the last fish diving down to the first fish releasing air again. We also estimated how many fish of the shoal took part in a breathing event. As the water turbidity did not allow counting of all individuals in the shoal but was sufficient to estimate roughly the fraction of the shoal that took a breath together, we scored whether <20%, 20–50%, 51%–80%, or >80% of the whole shoal took part in a breathing event. To still be able to compare individual breathing durations of individuals within the shoal to those obtained by the isolated subjects, we randomly picked 30 individuals that were well visible throughout the breathing event (by chance visible throughout the whole surfacing and diving succession) and scored the duration of their individual air-breathing release of old air (phase III) until diving back down from the surface (phase V), same as was scored for individual behavior (see above). Please note that both the estimation of the fraction of individuals that took part in the collective breathing as well as the random selection of the 30 individuals observed within the shoal for their breathing duration could be subject to some observer biases and inaccuracies (none-random choice of individuals observed, inaccuracy in estimating the fraction of partaking individuals).

### Statistics and reproducibility

To estimate how much behavioral variability we encounter in the individual breathing behavior, we calculated the Repeatability of the breathing behavior. This provides a measure of the among-individual variation both in breathing intervals as well as breathing durations (according to refs. ^79,80^). We used separate linear mixed models with trial (1st or 2nd) as a fixed factor to detect behavioral changes between the repeated test trials in intervals and durations, and included individual ID as a random subject factor. We used Pearson’s correlations to estimate whether breathing intervals and durations were correlated for each trial separately. Statistical tests were performed using SPSS 25 (IBM Inc.).

### The model of collective breathing

To simulate the observed collective air-breathing behavior, we use a stochastic integrate-and-fire (IF) framework proposed by Sarfati et al.^27^, which adapts the deterministic integrate-and-fire dynamics for self-sustained coupled oscillators originally introduced by Mirollo and Strogatz^81^. This framework allows inherently non-oscillating individuals (i.e., with irregular periods of activity when alone) to generate synchronized periodic behavior at the group level. Beyond analytical proofs, Sarfati et al.^27^ verified the model using empirical data from *P. carolinus* fireflies, demonstrating that individuals converge to a common interval between flashing bursts as the group size increases. It was assumed that all fireflies in isolation have the same distribution of inter-burst intervals. However, their analytical theory demonstrated that emergent periodicity and synchrony arise regardless of the specific distribution of the input intervals at the level of individual agents. This makes this framework well-suited for our study to model the synchronous surfacing behavior in a group of individuals with assumed consistent inter- and intra-individual variability of breathing patterns (see "Results").

Based on Sarfati et al.^27^, we simulated a group of *N* individual fish whose breathing dynamics are governed by the following Eq. (1):

1 $$\frac{d{V}_{i}}{{dt}}=\frac{1}{{T}_{{si}}}\left[1-{\varepsilon }_{i}(t)\right]-\frac{1}{{T}_{{di}}}{\varepsilon }_{i}(t)+{\sum }_{j=1,j\ne i}^{N}\frac{\beta }{N}{\delta }_{{ij}}{\varepsilon }_{j}(t)\left[1-{\varepsilon }_{i}(t)\right]$$

where variables *V*_*i*_ and *ε*_*i*_ characterize the internal state of an individual fish $i=\left\{1,...N\right\}$. The variable *V*_*i*_ represents an individual’s drive to come to the water surface to take a breath (‘motivation to surface’, see Fig. 1A), by analogy with the voltage or membrane potential in the IF of a biological neuron. It is an internal state that accumulates linearly over time until it reaches a threshold, ${V}_{{th}}=1$, triggering the action, i.e., taking a breath (“breathing event”, Fig. 1A). In other words, when an individual is not breathing, it “charges” its potential *V*_*i*_ to take a breath. This can be seen as an analogy to the decrease in blood oxygen levels that have been found to trigger air-breathing in fishes^34^. The values ${\varepsilon }_{i}(t)$ describe the binary state of the fish *i* at a time step *t*, i.e., *at the surface breathing* (discharging), ${\varepsilon }_{i}(t)=1$, or *underwater not breathing* (charging), ${\varepsilon }_{i}(t)=0$. The state ${\varepsilon }_{i}(t)$ is set to 1 as soon as the individual is fully charged (${V}_{i}=1$) and switches back to ${\varepsilon }_{i}(t)=0$ after completing the breath, i.e., when discharged to ${V}_{i}=0.$ The time ${T}_{{di}}$ is the breathing duration of an individual *i* and the time ${T}_{{si}}$ is the breathing interval between its two successive breathing events (Fig. 1B).

To explore how inter-individual differences influence the collective dynamics of air-breathing behavior, we consider each of the six experimentally derived distributions of individual air-breathing intervals obtained in isolation as a distinct breathing type (Fig. 2A). To simulate homogenous groups, we assigned the same breathing type to all individuals *i*, with ${T}_{{si}}$ values sampled from a single distribution. This will result in groups where individuals have the same breathing type. For heterogeneous groups, we considered that agents belong to different breathing types, such that we sampled ${T}_{{si}}$ values for each individual *i* from their own corresponding distributions. For the breathing duration, ${T}_{{di}}$, we sampled values directly from the experimental individual in the shoal breath duration distribution (see Fig. 3B). This way, both ${T}_{{si}}$ and ${T}_{{di}}$ are stochastic variables resampled for each agent *i* from the respective experimental distributions each time *t* the agent changes its state ${\varepsilon }_{i}(t)$. If an agent *i* is not breathing (${\varepsilon }_{i}(t)=0$) and detects other agents *j* at the water surface (i.e., breathing, ${\varepsilon }_{j}(t)=1$), its “charging” process is accelerated by a coupling strength of $\beta /{N}_{\beta }$, in proportion to the number of breathing fish *N*_*β*_ at a time *t* (Fig. 1C). As such, an agent *i* will reach the threshold ${V}_{{th}}$ faster, shortening its personal breathing time ${T}_{{si}}$ compared to when being alone.

We assumed global network connectivity (all-to-all) between individuals, i.e., ${\delta }_{{ij}}=1$ for any pair of individuals $(i,j)$, meaning that any agent can influence and be influenced by the other. Within this topology, we considered two coupling schemes: (1) homogeneous coupling, in which all individuals interact with identical coupling strength *β*, and (2) heterogeneous coupling, in which interaction strength depends on breathing type, with stronger intra-type coupling (${\beta }_{{intra}}$) among individuals of similar breathing types and weaker inter-type coupling (${\beta }_{{inter}}$) between individuals of different breathing types. Assuming that individuals self-assort within the shoal according to their breathing needs (refs. ^82,83^), stronger intra-coupling ${\beta }_{{intra}}$ reflects stronger interactions with agents in the immediate neighborhood, while weaker inter-coupling ${\beta }_{{inter}}$ corresponds to weaker interactions with far-away neighbors in the shoal. In this way, ${\beta }_{{intra}}$ and ${\beta }_{{inter}}$ provide a simple representation of stronger local interactions and weaker long-range interactions, reflecting behavioral self-assortment without assuming that individuals estimate others’ underlying breathing intervals.

Unlike Sarfati et al.^27^, we introduced a refractory period, during which an individual *i*, after finishing its breathing time ${T}_{{di}}$ (i.e., reaching ${V}_{i}=0$), remains socially unresponsive for the time ${\tau }_{{ref}}$. The refractory period is attributed to the stages where fish dive down from the surface and reorient into the shoal (see Fig. 1A, stages V-VI). A similar approach was used by Ramírez-Ávila et al.^84^ to model synchronization in *Photinus carolinus* by including silent (refractory) periods after a phase, but unlike those bursting, self-sustained oscillators, our units are stochastic and inherently non-periodic. Our exploration of the model revealed that a refractory period is required to generate the high degree of synchronization in a collective of stochastic agents employing our empirical distributions within the framework (see Supplementary Fig. S5A, Supplementary Note 3.3). To match the descriptive features of our experimental data (i.e., the distribution of collective breathing intervals and shoal partaking), we set a fixed refractory period of ${\tau }_{{ref}}=1.1$ s for the homogeneous coupling scheme and${\tau }_{{ref}}=2.1$ s for the heterogeneous coupling scheme (for details see Supplementary Note 3.3; Supplementary Figs. S5, S8).

We evaluated the performance of numerical simulations by statistically comparing the breathing interval distributions generated by the model (for different coupling strength values *β*) to the one experimentally obtained for the shoal (i.e., Laplace distribution, see Fig. 3C) using two-sided Kolmogorov-Smirnov tests. Particularly, the null hypothesis states that the simulation-generated samples follow the Laplace distribution with the same scale parameter as the experimental data (i.e, $b=3.55$). We do not fix the location parameter *μ* in the Laplace distribution, since according to Sarfati et al.^27^ the peak of the collective breathing interval distribution is expected to be determined by the shortest time between breathing events for an individual fish in isolation. Since the six individual distributions we use for the simulations represent a sub-sample of the entire shoal, we could have potentially missed the breathing types with such super-fast intervals, increasing the discrepancy in the comparison between distributions. To address this, we also test whether our model of synchronous breathing can reproduce a Laplace-like distribution in general, without imposing fixed parameters for the distribution.

When an individual synchronizes with others who have very different breathing patterns compared to its own, it has to adjust its individual natural rhythm more strongly than when synchronizing with those of a similar breathing type. We assume this mechanism would then promote spatial self-sorting within the shoal based on breathing type (forming “breathing clusters of similar breathing types”, see, for example, refs. ^82,83^). Due to self-sorting, spatial constraints would limit immediate interactions between individuals from different breathing clusters. Under these conditions, we expect to get partial synchronization (where only parts of the shoal participate in a synchronized breathing event) with *some degree* of compromise to emerge, which by definition will be less than in the case of full synchronization (where the whole shoal takes part in a breathing event = non-clustered heterogeneous shoals). To quantify how well the simulated partaking rates based on the above mechanism match our empirical observations of partaking rates, we computed the sum of squared differences (SSD) between the empirical probabilities of occurrence of each partaking category (i.e., “<20%,” “20–50%,” “50–80%,” “>80%”; see Fig. 3D), which represent the proportion of the shoal surfacing, and the corresponding probabilities from the simulation. Furthermore, we analyzed the level of individual compromise for simulated homogenous, heterogeneous with varying degrees of clustering, and non-clustered heterogeneous groups in relation to the fraction of simultaneously breathing individuals (Fig. 7). Individual compromise is calculated for each breathing event as the difference between an individual’s simulated breathing interval in a group and the individual’s empirical average breathing interval when alone (in seconds).

### Quantification of group synchrony and collective breathing events

To quantify group synchronization in the simulations, we used the *spike_contrast* function from the *Elephant.spike_train_synchrony* library in Python^85^. For each individual in the group, we constructed a spike train which represents an array of the starting times of an individual’s *i* breathing events (i.e., when ${\varepsilon }_{i}(t)=1$) over the duration of a single simulation (i.e., 10,000 time steps). The *spike_contrast* function is then applied to the set of individual spike trains of all agents to compute a group-level synchrony index.

To measure collective breathing intervals in the simulations, the model outputs a binary matrix *ℇ*_*N*×*T*_ where each row corresponds to an individual and each column to a simulation time step (${T=n}_{{steps}}$). Matrix entries *ℇ*_*i,t*_ take the value 1, if an individual *i* is breathing at a given time step *t*, and 0 otherwise. Group-level breathing activity ${{\mathcal{A}}}\left(t\right)$ at each time step *t* is computed as the sum of the column values divided by the total number of agents N, i.e., ${{\mathscr{A}}}\left(t\right)=\frac{1}{N} \sum_{i=1}^{N}{{{\mathscr{E}}}}_{i,t}$. This way, ${{\mathscr{A}}}\left(t\right)=1$ indicates full synchrony (all individuals breathing simultaneously), while intermediate values correspond to partial participation. To identify the onset of collective breathing events, we apply the *find_peaks* function (from Python SciPy library) to the group activity signal ${{\mathscr{A}}}\left(t\right)$ with parameters of height = 0.1 and distance = 200. The height threshold ensures that at least 10% of the group is breathing simultaneously, while the distance parameter requires detected peaks to be separated by at least 200 time steps (i.e., 2 s), preventing multiple detections of the same event. If ${\left\{{t}_{k}\right\}}_{k=1}^{K}$ denote the time points of the detected peaks in ${{\mathscr{A}}}\left(t\right)$, i.e., corresponding to the collective breathing events, then collective breathing intervals are defined as ${\triangle }_{k}={t}_{k+1}-{t}_{k}$, and the fraction of the group participating in event *k* is given by ${{\mathscr{A}}}\left({t}_{k}\right)$.

### Fitting probability distributions to empirical breathing intervals

We used Python statistical packages **SciPy** and the **Fitter** library to systematically identify candidate distributions for the breathing intervals data in Fig. 2A, C and Fig. 3C. **SciPy** provides over 80 continuous probability distributions, and the **Fitter** class scans all of these distributions by estimating parameters via maximum likelihood, excluding those for which fitting fails, and ranking the remaining candidates based on the sum of squared errors between the empirical data and fitted probability density functions.

We used this procedure for initial exploratory analysis to fit individual breathing intervals data in Fig. 2A, from which six candidate distributions were identified for further consideration: exponential, Weibull, gamma, normal, log-normal, and Rayleigh. We also included the uniform distribution as a simple baseline model to assess if individual breathing intervals exhibit structured patterns, rather than being evenly random across the observed range. For each candidate distribution, we estimated parameters of the fit using the SciPy stats.fit function and compared the fitted distributions with the empirical data using the Kolmogorov-Smirnov test. We report the resulting KS statistics and p-values in Tables S2 and S3. Based on the KS statistic (Table S2), the Gamma distribution shows the closest agreement (i.e., the smallest KS statistic) with the empirical distributions of breathing intervals among the considered distribution candidates for each individual. For individual 2, however, the Normal distribution yields the smallest KS statistic, but we selected the Gamma distribution for consistency across individuals, under the assumption that the individual’s breathing intervals can be represented by a single distribution type.

We also applied the same procedure to find out the fits to the combined individual breathing interval data from trials 1 and 2 in Fig. 2C, and report the resulting KS statistics and p-values in Tables S4 and S5. Based on the KS statistics, the Gamma distribution yields the smallest values in both trials (Table S4), indicating the closest agreement with the empirical data among the tested distributions. While several distributions show similar KS statistics in trial 2, we selected the one with the lowest value, i.e., Gamma distribution.

The exploratory fitting analysis for collective breathing intervals in Fig. 3C identified several candidate distributions, among which the Laplace distribution has the smallest sum of squared errors with the empirical data (see Table S6). Based on this, we selected the Laplace distribution as the most suitable candidate and estimated its parameters using the **curve_fit** function from Python **SciPy** package, which optimizes the parameters $(\mu ,b)$ of the fitted function (see Eq. 2) by minimizing the sum of squares of residuals with respect to the empirical data.

2 $${Laplace}\left(x\right){{\rm{:= }}}\frac{1}{2b}{e}^{-\frac{\left|x-\mu \right|}{b}}$$

The distribution fitting and KS statistics were computed from the raw data and were therefore independent of histogram binning. For histograms of collective breathing intervals and the combined breathing intervals individuals as well as their breath durations (see Fig. 3A, B), the Freedman-Diaconis rule^86^ was used to determine an optimal global bin width (3.83 s for Fig. 3A; 0.08 s for Fig. 3B). The resulting bin widths were used for respective distributions within the corresponding range to ensure consistent binning across conditions (i.e., within each subpanel).

Histograms of individual breathing intervals when in isolation (Fig. 2A) were constructed using a manually selected number of bins within each individual’s data range. This approach was chosen to account for differences in sample size and variability between individuals for better visualization of individual-specific distributional features. Specifically, histograms of individual breathing intervals (Fig. 2A) were constructed using 22 bins for individual 1 and 15 bins for the remaining individuals. For individual breath durations (Fig. 2B), 10 bins were used for all individuals. For the combined breathing intervals and breath durations across trials 1 and 2 (Fig. 2C, D), bin widths were manually adjusted for visual clarity and set to 9 s (Fig. 2C) and 0.04 s (Fig. 2D), respectively.

### Reporting summary

Further information on research design is available in the Nature Portfolio Reporting Summary linked to this article.

## Supplementary information

Supplementary Information

Description of Additional Supplementary Files

Supplementary Data 1

Supplementary Video 1

Supplementary Video 2

Reporting Summary

## Data Availability

The numerical source data underlying Figs. 2–3 are provided in Supplementary Data 1. The simulation source data underlying Figs. 4–7 are available in the Zenodo repository at https://zenodo.org/records/19813940^87^.

## Code availability

The custom recording software^78^ used in this study is available in the Github repository at https://github.com/fritzfrancisco/ArapaimaBehaviour.
