# Supplementary material for: Air-breathing synchrony in juvenile Arapaima gigas reveals collective coordination under individual physiological constraints
Source: Commun Biol. 2026 Jun 17;9:831. doi: 10.1038/s42003-026-10472-w (PMC13276053; doi:10.1038/s42003-026-10472-w)
Supplement: Supplementary file 1 — Supplementary Information [file 42003_2026_10472_MOESM1_ESM.pdf]

Supplementary Materials

Air-breathing synchrony in juvenile *Arapaima gigas* reveals collective coordination under individual physiological constraints

Palina Bartashevich[1,2,#], Fritz A. Francisco[3], Alessandra Escurra-Alegre[1,2,4,5], Fabian Schäfer[4], Sven Wuertz[4], Jens Krause[1,2,4], Werner Kloas[1,4,5], David Bierbach[1,2,4#]

1 Faculty of Life Sciences, Albrecht Daniel Thaer-Institute of Agricultural and Horticultural Sciences, Humboldt-Universität zu Berlin, Invalidenstrasse 42, 10115 Berlin, Germany
2 Cluster of Excellence 'Science of Intelligence', Technical University of Berlin, Marchstrasse 23, 10587 Berlin, Germany
3 Department of Biology, University of Massachusetts Boston, 100 Morrissey Blvd, Boston, MA 02125, USA
4 Department of Fish Biology, Fisheries and Aquaculture, Leibniz-Institute of Freshwater Ecology and Inland Fisheries, Müggelseedamm 310, 12587 Berlin, Germany
5 Faculty of Life Sciences, Institute of Biology, Humboldt-Universität zu Berlin,10099 Berlin, Germany

# corresponding authors: PB: bartashevich.palina@gmx.de; DB david.bierbach@hu-berlin.de

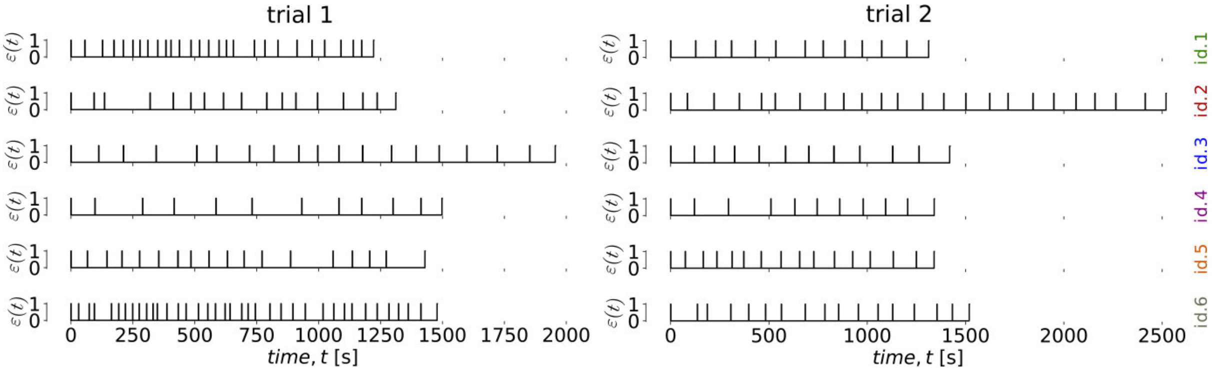

Fig. S1. Time series of the individual breathing behavior of six individual fish recorded in isolation. Each spike, i.e., $\varepsilon(t) = 1$, depicts a breathing event of a single fish.

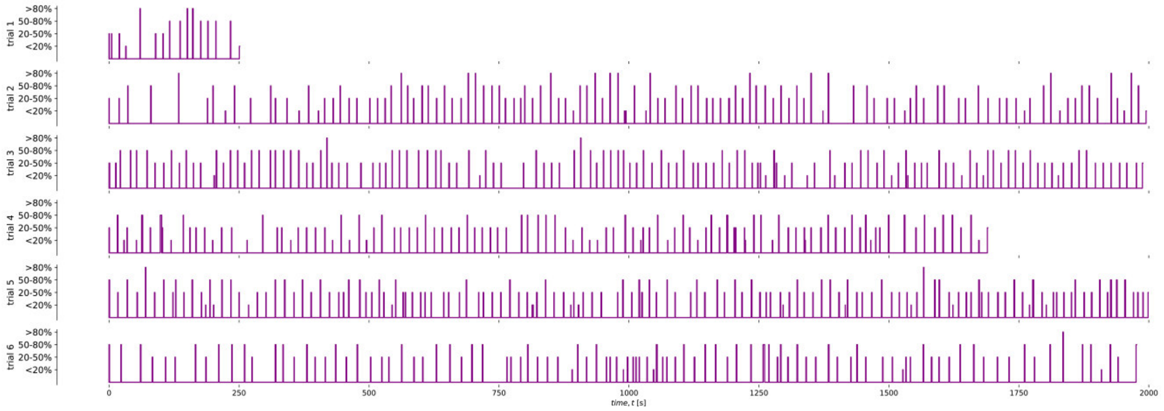

Fig. S2. Time series of the collective breathing behavior of the investigated Arapaima shoal. Each spike depicts a collective breathing event, with a certain proportion of the shoal partaking (estimated as <20%, 20-50%, 50-80%, and >80% of the shoal).

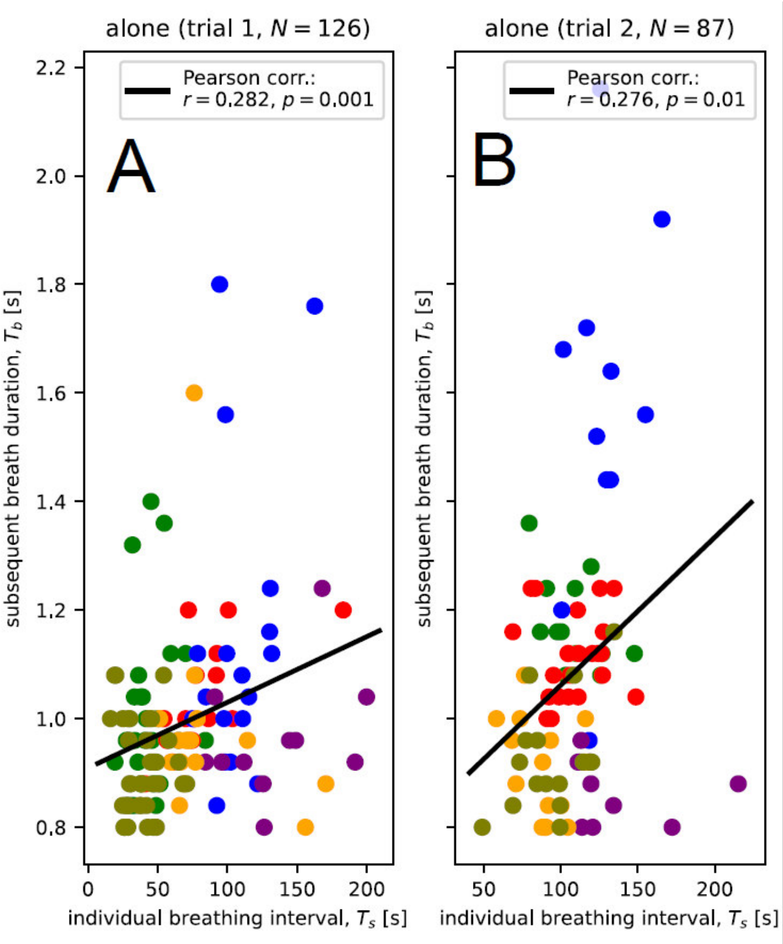

Fig. S3. Scatter plot of individual breathing intervals in isolation versus subsequent breath durations, with a fitted regression line for (A) trial 1 and (B) trial 2. Dots of the same color correspond to the same individual. The positive correlation in both trials (trial 1: r=0.282, P<0.00; trial 2: N=87, r=0.276, P=0.01) suggests that a longer time since the last breath is associated with a longer subsequent breathing duration.

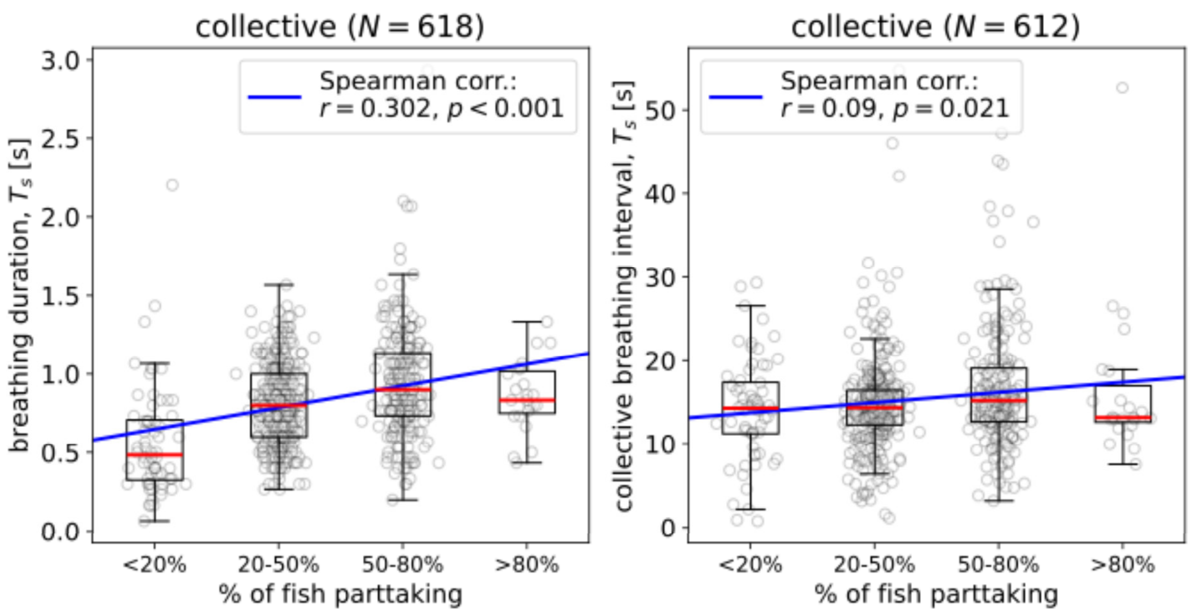

Fig. S4. Scatter and box plots showing the relationship between the proportion of the shoal partaking in the breathing event and (A) the breathing duration, (B) the next collective breathing interval. The positive correlation (Spearman's rank coefficient $r_{\text{duration-partaking}} = 0.302$, $p<0.001$) in (A) suggests that more fish taking part in the breathing event is associated with a longer breathing duration. The low Spearman's rank coefficient ($r_{\text{interval-partaking}} = 0.09$, $p=0.021$) in (B) indicates that there is little to monotonic relationship between the proportion of the shoal partaking and the subsequent collective breathing interval. The red horizontal lines in the boxplots indicate the respective medians; the boxes show the interquartile range and the whiskers extend to 1.5×IQR.

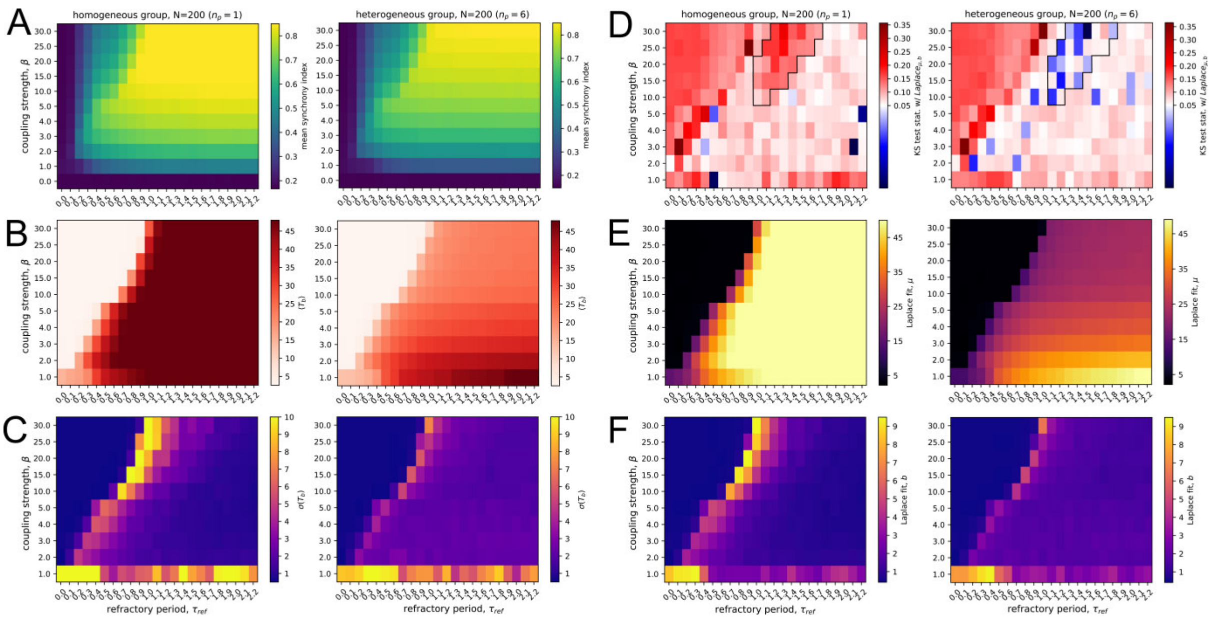

Fig. S5. Impact of the refractory period τ_{ref} and coupling strength β on collective breathing patterns' characteristics in simulated homogenous and heterogeneous groups (N=200). (A) Mean synchrony index of breathing events. (B) The mean and (C) standard deviation of the simulated collective breathing intervals. (D) Likelihood of encountering a Laplace-like distribution of collective breathing intervals. The KS-test values above 0.05 (in red) indicate rejection of the null hypothesis, meaning the collective breathing intervals of the simulated group do not follow a Laplace distribution. (E) Estimated location (peak/center) and (F) scale (spread) parameters of Laplace distributions fitted to the simulated collective breathing intervals. The results were generated from 20 simulations, 1000 time steps each with $dt = 0.01$. Homogeneous groups include individuals of one breathing type (id=4), while heterogeneous groups include individuals from six distinct breathing types.

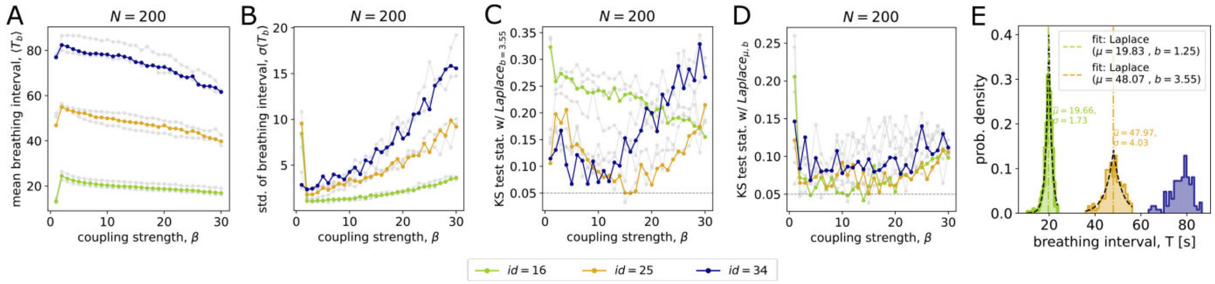

Fig. S6. Simulated semi-heterogeneous groups, each composed of the individuals of two breathing types (group id=16 in green: with 100 agents of id=1 and 100 agents of id=6; group id=25 in yellow: with 100 agents of id=2 and id=5; group id=34 in blue: with 100 agents of id=3 and 100 agents of id=4). (A) Mean and (B) standard deviation of the collective breathing intervals depending on the strength of social coupling β. Two-sided Kolmogorov-Smirnov test results verify at each β whether breathing intervals in the modelled shoal come from (C) the Laplace distribution with the empirical scale parameter $b = 3.55$ or from (D) any Laplace-like distribution without fixed parameters. (E) Depiction of the model-generated collective breathing interval distributions for three semi-heterogenous independent groups with β parameters corresponding to their best fits to the Laplace distribution (β=14 for a group with id=16 and β=15 for groups with ids ∈(25, 34)). The results in gray color in (A-D) correspond to homogeneous group compositions (for details see Fig. 4B-E in the main text) and are depicted for comparison.

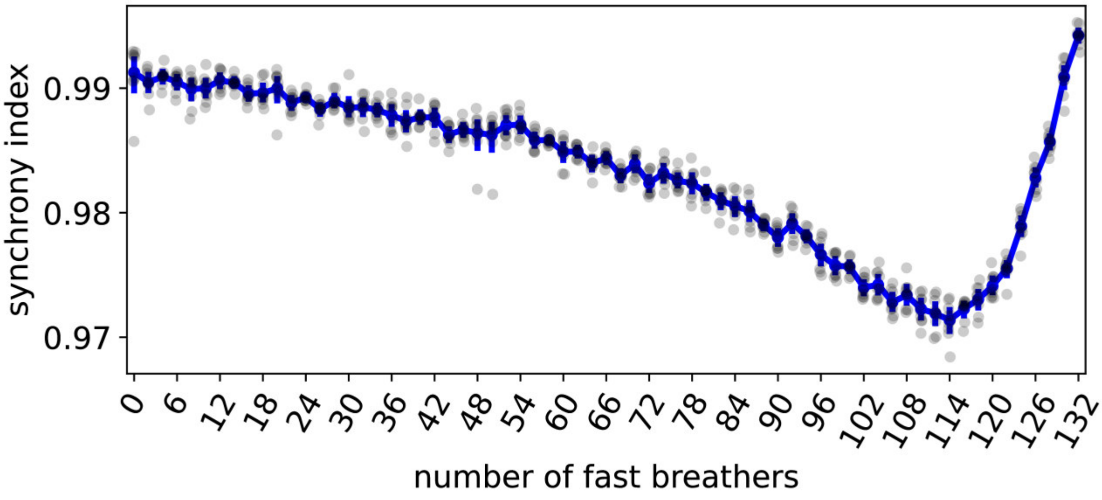

Fig. S7. Impact of the number of fast breathers on the collective breathing synchrony in a simulated group of N=200. The number of present individuals of each of the breathing types (“fast”, “medium”, and “slow”) is re-calculated as (N_{fast}, ⅓*N, ⅔*$N - N_{fast}$), respectively. This way, the proportion of the medium breathers is kept constant to 34%, while the proportions of fast and slow breathers vary. The results were generated from 10 simulations, 1000 time steps each with $dt = 0.01$, social coupling strength of $\beta = 14$ and the refractory period $\tau_{ref} = 1.1$. The mean synchrony index decreases as the number of fast breathers increases from 0 up to 114 (57% of the group, with 8% slow breathers) but increases afterwards, reaching full synchrony at 132 fast individuals (66% the group, with no slow breathers). Overall, synchrony of individual breathing events remains high within the range of 0.97 to 1. Error bars represent standard deviation.

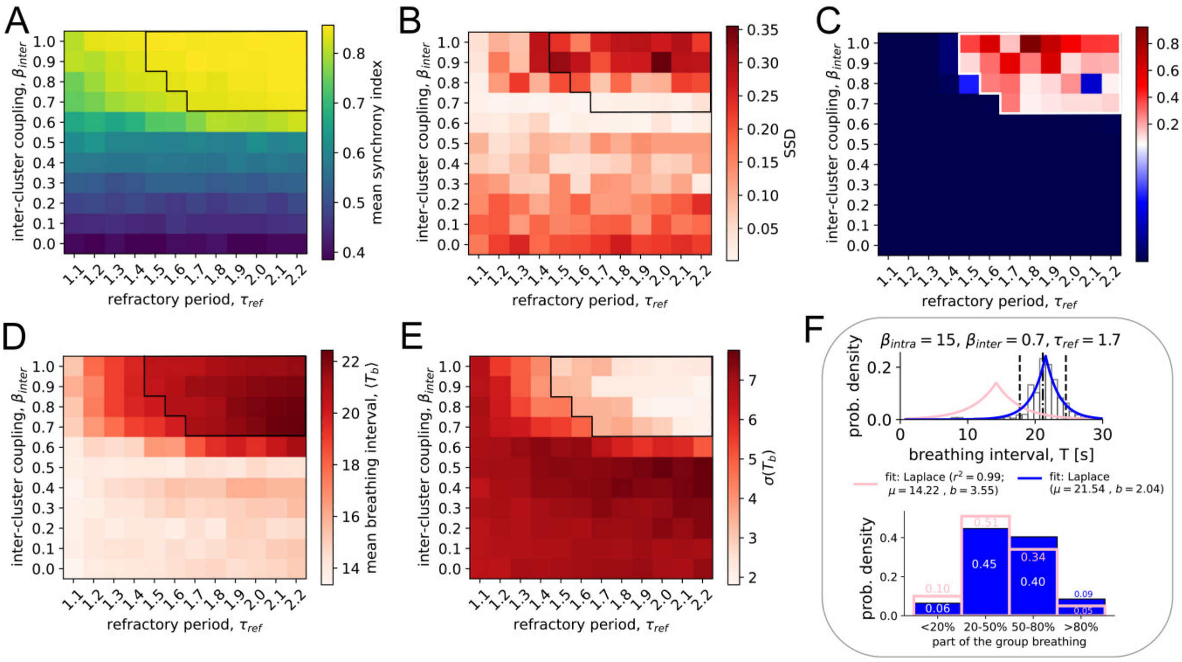

Fig. S8. Impact of the refractory period τ_{ref} on simulated collective breathing patterns' characteristics depending on the inter-cluster coupling strength $\beta_{inter_{clu}}$. (A) Mean synchrony index of breathing events. (B) The sum of squared differences (SSD) between empirical and simulation-generated probabilities of the proportions of individuals participating in breathing events. (C) Likelihood of encountering a Laplace-like distribution of collective breathing intervals. If the p-value is below 0.05 (in blue), the intervals do not follow a Laplace-like distribution. (D) The mean and (E) the standard deviation of the simulated collective breathing intervals. (F) Histograms of the distributions of collective breathing intervals and proportions of the group participating in the breathing event in simulation (in blue) versus empirical data (in pink) with $\beta_{inter} = 0.7$ and $\tau_{ref} = 1.7$s. The results were obtained from simulations of heterogeneous groups ($N = 200$) consisting of individuals of three breathing types ($ids \in (4,5,6)$), over 20 realizations of 1000 time steps each with $dt = 0.01$ and fixed intra-cluster coupling strength of $\beta = 15$. Based on the results in the main text, we assumed fast breathers are in the minority and represent the group with an unequal ratio of fast:medium:slow breathers as 1:2:2, corresponding to cluster sizes of $N_{clu} \in (40,80,80)$ agents.

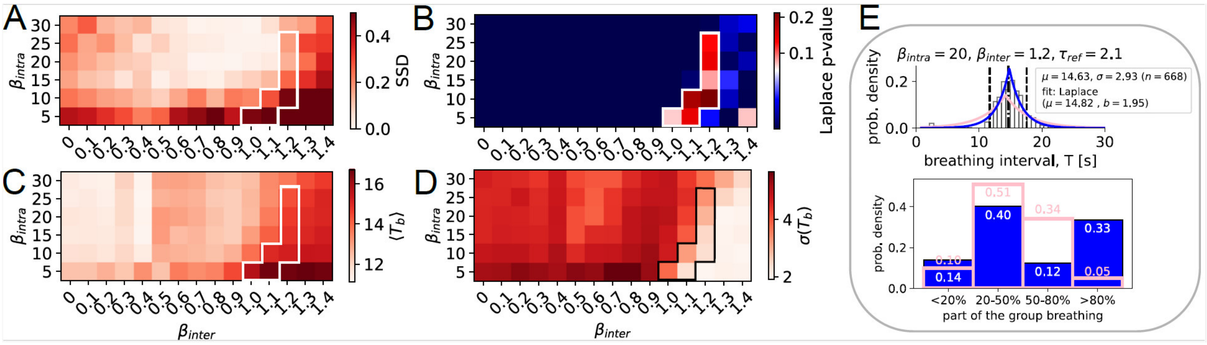

Fig. S9. Influence of including a modified fastest breathing individual type on the collective breathing interval distribution and partaking rates in simulated heterogeneous groups ('proof-of-concept'). (A) Difference between empirical and simulation-generated probabilities of the proportions of individuals participating in breathing events depicted as the sum of squared differences (SSD) for varying intra- and inter-cluster coupling strengths. (B) Likelihood of encountering a Laplace-like distribution for different combinations of the intra- and inter-cluster coupling strengths based on the KS test statistics. If the p-value is below 0.05 (in blue), the intervals do not follow a Laplace-like distribution. (C) The mean and (D) standard deviation of the simulation-generated collective breathing intervals for varying intra- and inter-cluster coupling strengths. (E) Histograms of the distributions of collective breathing intervals and proportions of the group participating in the breathing event in simulation (in blue) versus empirical data (in pink) for the best parameter match. Simulated groups were composed of $N = 200$ individuals from three breathing types with $ids \in (7,5,4)$, where type $id = 7$ was obtained by shifting the empirically fastest breathing distribution ($id = 6$) by −7s. Fast breathers (with $= 7$) were in the minority, with a fast:medium:slow ratio of 1:2:2, corresponding to cluster sizes of $N_{clu} \in (40,80,80)$ agents.

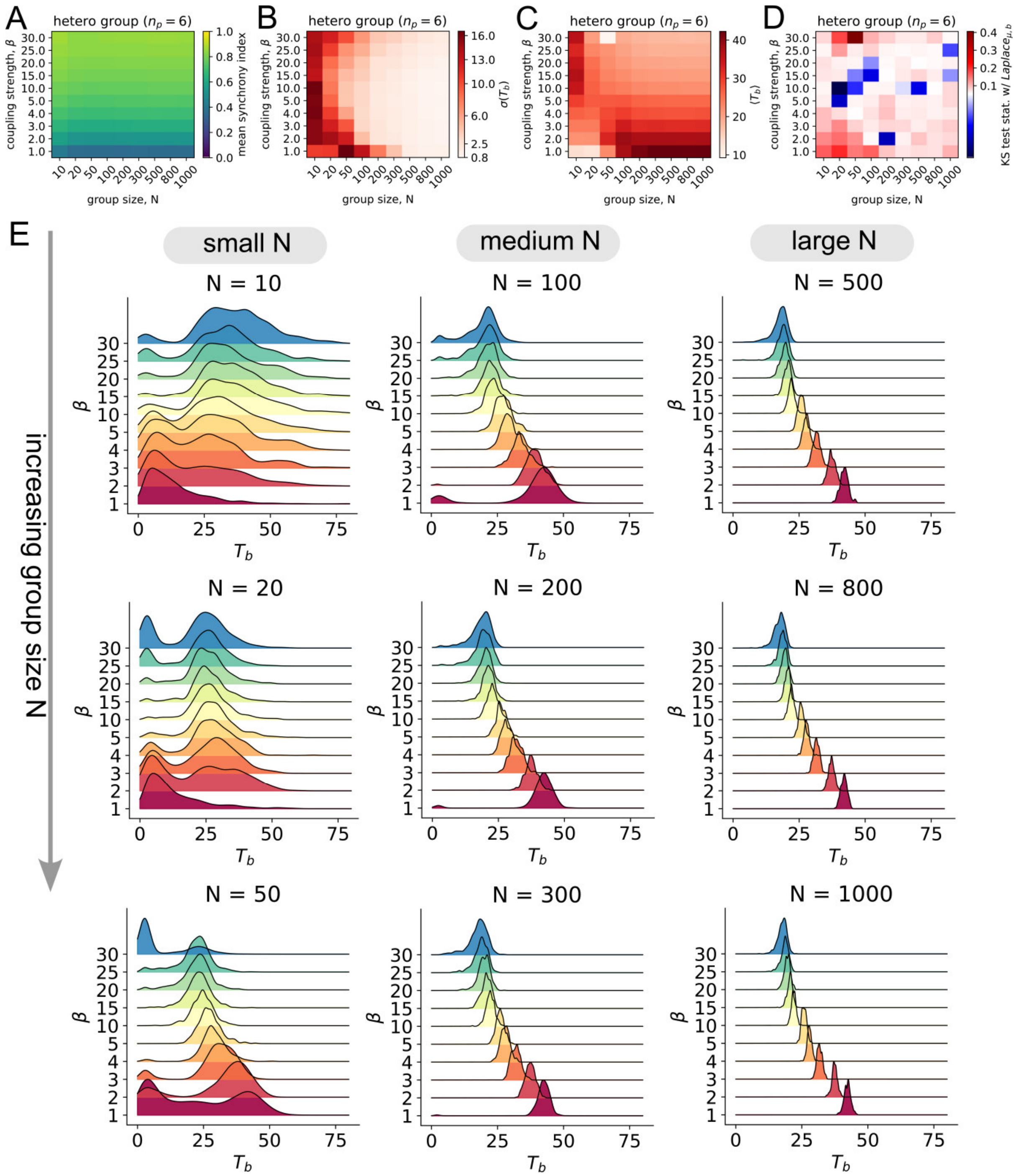

Fig. S10. Impact of group size N and coupling strength β on collective breathing interval distributions in simulated heterogeneous groups. (A) Mean synchrony index of breathing events across different N and β combinations. (B) Variability (standard deviation) and (C) mean of emergent collective breathing intervals by group size and coupling strength. (D) Two-sided Kolmogorov-Smirnov tests assess whether breathing intervals at each $N - \beta$ combination follow any Laplace-like distribution without fixed parameters. If the KS statistic value is below 0.05 (in blue), the intervals follow a Laplace-like distribution. (E) Illustration of collective breathing intervals distributions for each group size N across coupling strengths β∈{1,2,3,4,5,10,15,20,25,30}. Small groups show high variability and bimodal distributions of

collective breathing intervals, while medium and large groups display a single peak that shifts to shorter intervals as β increases.

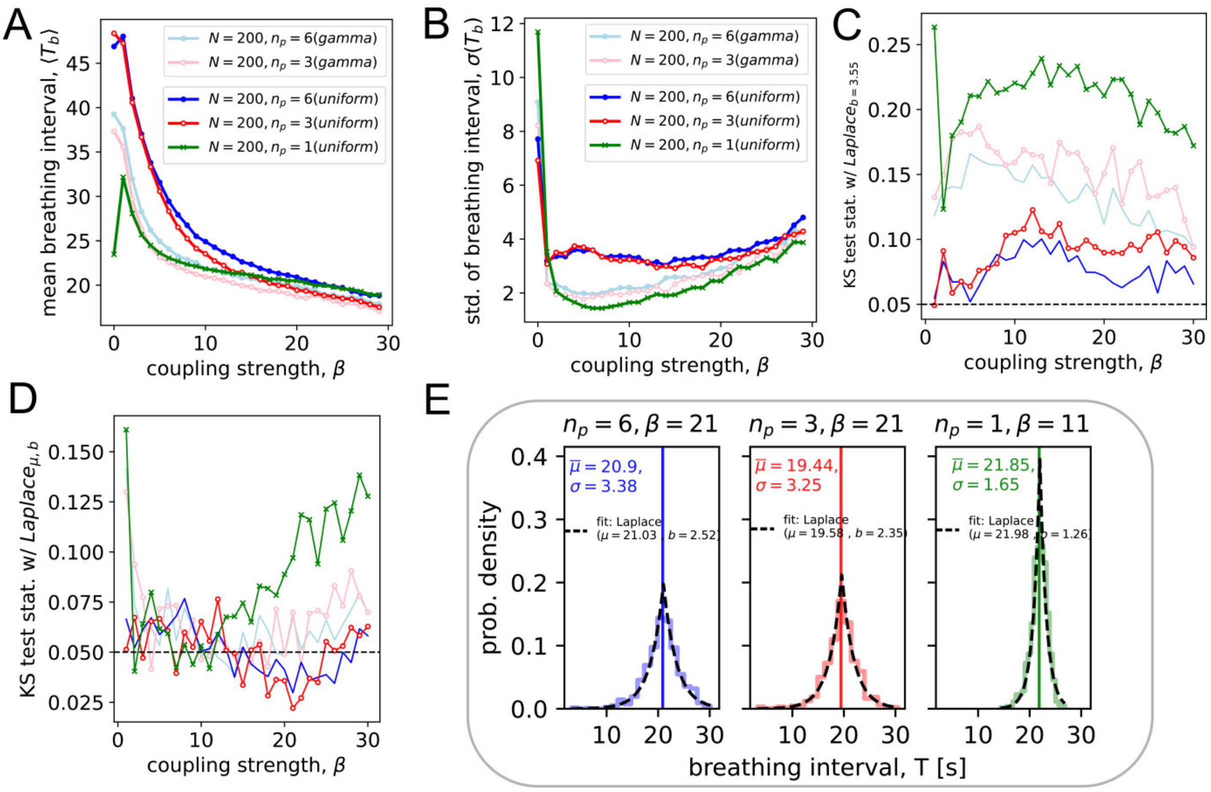

Fig. S11. Effect of individual breathing interval distributions (uniform vs. gamma) on collective air-breathing behavior in simulated homogeneous and heterogeneous groups. (A) Simulated mean breathing intervals and (B) their standard deviation for heterogeneous groups composed of either 6 (in blue) or 3 (in red) individual breathing types, and for a homogeneous group with a single breathing type (in green). (C) Two-sided Kolmogorov-Smirnov test results verify at each β whether breathing intervals in the modelled groups come from the Laplace distribution with the empirical scale parameter $b = 3.55$ or (D) from any Laplace-like distribution without fixed parameters. The KS-test values above 0.05 indicate rejection of the null hypothesis, meaning the collective breathing intervals of the simulated group do not follow a Laplace distribution. (E) Model-generated collective breathing interval distributions resulting from uniformly distributed individual breathing intervals in a heterogeneous group of N=200 individuals with 6 and 3 breathing types, and in a homogeneous group, shown for β parameters corresponding to their best fits to the Laplace distribution.

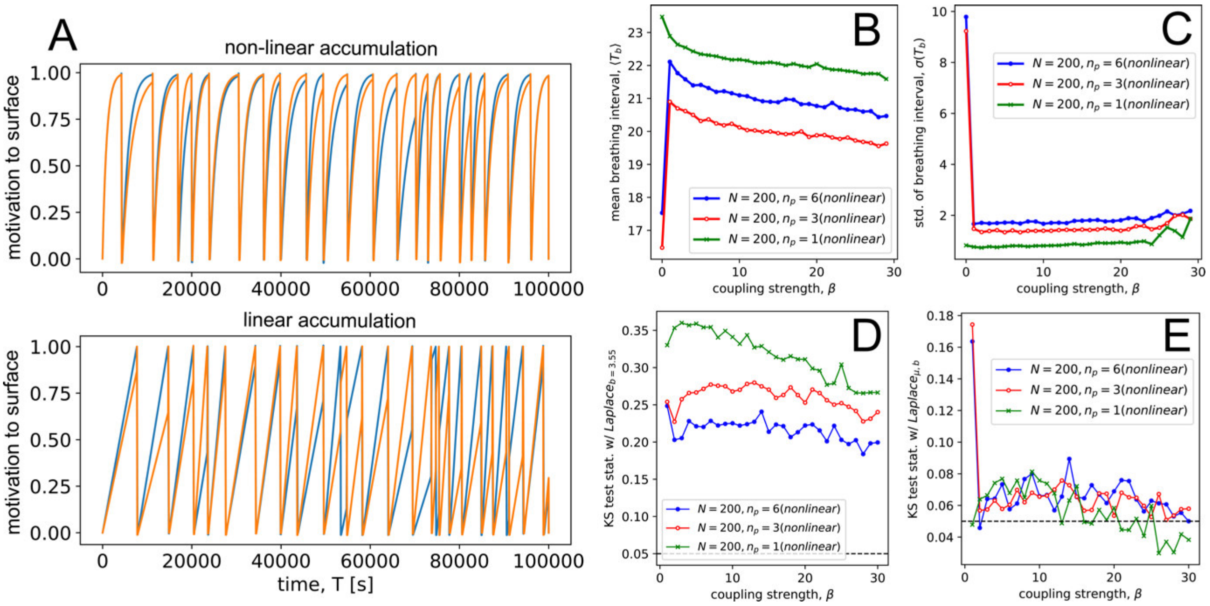

Fig. S12. Impact of non-linear, concave-down individual accumulation rate on the emergent collective dynamics. (A) Illustrative example of concave-down (non-linear) and linear individual accumulation dynamics for two interacting individuals (in orange and in blue) according to Eq. (S2) and Eq. (1), respectively. (B) Simulated mean breathing intervals and (C) their standard deviation for homogeneous $(n_p = 1)$ and heterogeneous groups composed of either only $n_p = 3$ or all $n_p = 6$ different individual breathing types for group sizes of $N = 200$. (D) KS test results verify at each β whether breathing intervals in the modelled groups come from the Laplace distribution with the empirical scale parameter $b = 3.55$ or (E) from any Laplace-like distribution without fixed parameters. All distributions were generated from 10 simulations, 1000 time steps each with $dt = 0.01$ and the refractory period $\tau_{ref} = 1.1$.

Table S1. Proportion of simulated breathing events where individuals of certain type (i.e., "slow", "medium", "fast" breather) were breathing alone without synchronizing with a collective. The results are reported as a function of inter-cluster coupling strength $\beta_{inter_{clu}}$ for different breathing types. The proportion is computed relative to the total number of breathing events from 10 simulations, 1000 time steps each with $dt = 0.01$, intra-cluster coupling strength of $\beta_{intra_{clu}} = 15$ and refractory period $\tau_{ref} = 2.1$. The values correspond to the fraction of data points in Fig. 6 of the main text where the breathing group ratio equals 0 (i.e., the individual took breath alone) relative to the total number of points (breathing events) for the corresponding $\beta_{intra_{clu}}$. The values in red are in the transition region, where the collective breathing interval distribution best matches the Laplace-like distribution and empirical shoal partaking rates.

$\beta_{inter_{clu}}$	slow breather	medium breather	fast breather
**0.0**	-	0.01	0.05
**0.1**	0.06	0.01	0.06
**0.2**	0.03	0.02	0.07
**0.3**	0.05	0.01	0.11
**0.4**	0.07	0.01	0.12
**0.5**	0.09	0.09	0.13
**0.6**	0.01	0.09	0.32
**0.7**	-	0.01	0.17
**0.8**	-	0.01	0.04
**0.9**	-	-	0.01
**1.0**	-	-	0.004
**1.5**	-	-	-
**2.0**	-	-	0.03
**2.2**	-	-	0.12
**2.5**	-	-	0.40
**2.8**	-	-	0.57
**3.0**	-	-	0.32
**4.0**	-	-	0.01
**5.0**	-	-	0.002
**15.0**	-	-	0.002

Table S2. Kolmogorov–Smirnov (KS) statistics for different fitted distributions for each of the six individual breathing interval samples. The smallest KS statistic in each column (in bold) indicates closer agreement between the fitted distribution and the empirical data.

	ind1	ind2	ind3	ind4	ind5	ind6
**Exponential**	0.133	0.263	0.256	0.274	0.241	0.140
**Weibull**	0.092	0.086	0.111	0.162	0.737	0.103
**Gamma**	**0.082**	0.083	**0.105**	**0.158**	**0.137**	**0.092**
**Normal**	0.181	**0.073**	0.130	0.207	0.207	0.197
**Log-normal**	0.096	0.114	0.109	0.165	0.173	0.098
**Rayleigh**	0.180	0.124	0.109	0.187	0.213	0.187
**Uniform**	0.289	0.313	0.262	0.297	0.428	0. 336

Table S3. Kolmogorov–Smirnov (KS) test p-values for different fitted distributions for each of the six individual breathing interval samples. The largest value in each column (in bold) indicates closer agreement between the fitted distribution and the empirical data.

	ind1	ind2	ind3	ind4	ind5	ind6
**Exponential**	0.447	0.007	0.036	0.070	0.040	0.229
**Weibull**	0.863	0.911	0.832	0.583	2.4e-18	0.592
**Gamma**	**0.934**	0.930	**0.876**	**0.615**	**0.542**	**0.723**
**Normal**	0.138	**0.976**	0.663	0.285	0.112	0.028
**Log-normal**	0.827	0.651	0.847	0.564	0.261	0.657
**Rayleigh**	0.142	0.547	0.841	0.404	0.094	0.043
**Uniform**	0.002	0.001	0.030	0.038	7.6e-06	7.5e-06

Table S4. Kolmogorov–Smirnov (KS) statistics for differently fitted distributions for the combined individual breathing interval data across trials. For each trial, the smallest value (in bold) indicates the distribution with the closest agreement to the empirical data among the tested candidates.

	Trial1	Trial2
**Exponential**	0.125	0.296
**Weibull**	0.453	0.079
**Gamma**	**0.045**	**0.074**
**Normal**	0.117	0.079
**Log-normal**	0.052	0.076
**Rayleigh**	0.122	0.122

Table S5. Kolmogorov–Smirnov (KS) test p-values for differently fitted distributions for the combined individual breathing interval data across two trials. For each trial, the largest value (in bold) indicates the distribution with the closest agreement to the empirical data among the tested candidates.

	Trial1	Trial2
**Exponential**	0.035	2.9e-07
**Weibull**	3.8e-24	0.621
**Gamma**	**0.954**	**0.700**
**Normal**	0.058	0.623
**Log-normal**	0.867	0.673
**Rayleigh**	0.042	0.140

Table S6. Goodness-of-fit metrics returned by the Python *Fitter* library for candidate distributions fitted to the collective breathing interval data. Distributions are ranked based on the total squared difference between the empirical density and the fitted distribution's density (sum of squared errors, SSE).

	SSE	aic	bic	ks_statistic	ks_pvalue
**laplace**	0.0089	1225.085	1233.918	0.0528	0.0632
**loglaplace**	0.0090	1064.145	1077.395	0.0426	0.2098
**asymmetric laplace**	0.0099	1146.107	1159.357	0.0533	0.0594
**gamma**	0.0254	1238.095	1251.345	0.1021	0.0000
**exponential**	inf	inf	inf	NaN	NaN

Supplementary Note 1: Agent-based simulations with homogenous group compositions.

Using computer simulations, we found that collective breathing intervals in homogeneous groups did not fit a Laplace distribution with the empirical scale parameter b, except for a group composition with individuals of id=2 (see in the main text, Fig. 4D). While homogeneous groups of id=2 with high coupling strength $\beta = 15$ resembled the empirical collective breathing interval distribution in shape, a high mean and location parameter ($\mu = 44.79$) indicated a significant deviation from the experimental data (see in the main text, Fig. 4A, F). Homogeneous groups of id=1 or id=6 were both described by smaller mean of breathing intervals compared to others, but did not fit the Laplace distribution of collective breathing intervals with the empirical scale parameter (see in the main text Fig. 4A, D). For instance, homogenous groups of id=1 generated Laplace-like distributions of collective breathing intervals with location parameter of $\mu = 22.18$ but had a narrower shape ($b = 1.11$) than the experimental distribution (see in the main text Fig. 4E, F). Meanwhile, the breathing patterns of other homogeneous group compositions did not reproduce a Laplace-like distribution of collective breathing intervals in general (Fig. 4E).

Supplementary Note 2: Agent-based simulations with semi-heterogeneous group compositions.

As a special case of heterogenous group composition, we analyzed the collective breathing dynamics of semi-heterogeneous groups. Each semi-heterogenous group (N=200) consisted of an equal proportion of individuals from two breathing types which characterized by close mean breathing intervals: group (id=16) with 100 individuals of id=1 and 100 individuals of id=6; group (id=25) with 100 individuals of id=2 and 100 individuals of id=5; group (id=34) with 100 individuals of id=3 and 100 individuals of id=4. We found that semi-heterogeneous compositions closely resemble the collective dynamics of their homogeneous counterparts (see Fig. S6A-C). This suggests that the inter-individual breathing differences between the constituting types within each composition (although statistically consistently differ for single individuals in isolation) are not strong enough to significantly impact group behaviour. This may also imply that, while breathing types vary on a continuous scale, the most impact on collective breathing dynamics comes from “category-like" differences between individuals. Overall, the results showed that the mean breathing interval in semi-heterogeneous groups was shifted towards the faster breathing type (Fig. S6A, Fig. S6E vs. Fig. 4F in the main text).

Notably, when a breathing type that followed a Laplace distribution of the breathing intervals in a homogeneous shoal (e.g., id = 1 or id =2, see Fig. 4E-F in the main text) was mixed with another breathing type that did not follow a Laplace distribution in a homogeneous shoal, the collective breathing interval distribution of such a two breathing types mixed group remained Laplace (see e.g., id=25 or id=16 in Fig. S6D-E). This suggests that a subgroup (N=100) of a breathing type that followed a Laplace distribution of breathing intervals in a homogeneous case remains robust and even dominates, i.e., shapes the overall group breathing dynamics, when mixed with a close, yet distinct, breathing type. Indeed, a semi-heterogeneous group (id=25) with individuals of id=2 and id=5 followed a shape of empirical Laplace distribution (see Fig. S6C in orange, coupling strength $\beta = 15$), similarly to a homogenous group consisting of only individuals of id=2 (see in the main text Fig. 4D in red). Other semi-heterogeneous group compositions (id=16 and id=34) did not fit the Laplace distribution with the empirical scale parameter b (Fig. S6C), similarly to their homogeneous counterparts (see in the main text Fig. 4D).

Supplementary Note 3: Model robustness analysis.

Supplementary Note 3.1. Robustness to the shape of individual breathing interval distributions.

We tested the robustness of the Laplace-like collective breathing intervals to the shape of individual distributions. Although the Kolmogorov-Smirnov (KS) test indicated that a uniform distribution poorly fits the empirical individual breathing interval data (Tables S1-S2), we assessed the model's sensitivity to the assumption that individual breathing intervals could be uniformly distributed. Additional simulations (Fig. S11) were performed in which individual breathing intervals were sampled from six uniform distributions defined over each individual's empirical range.

Under this assumption, heterogeneous groups (N = 200) with either $n_p = 6$ or $n_p = 3$ breathing types still produced a Laplace distribution at sufficiently strong coupling (β > 15; Fig. S11D,E), consistent with results obtained using gamma-distributed intervals (Fig. 4J,K). The mean collective breathing interval decreased with increasing coupling strength and converged to similar values at larger β (Fig. S11A).

At moderate coupling strengths (i.e., $\beta > 1$, see Fig. S11B), sampling individual breathing intervals from heterogeneous uniform distributions leads to greater variability in the emergent collective breathing intervals than in the gamma case, which in turn results in a smaller KS statistic when compared to the empirical Laplace distribution (Fig. S11C). Nevertheless, neither case provides a good match to the empirical Laplace distribution (Fig. S11C). Notably, homogeneous population with uniform individual sampling does not produce any Laplace distribution of collective breathing intervals for stronger coupling ($\beta > 11$, see Fig. S11D), consistent with the behavior observed for gamma-distributed individual intervals (see main text Fig. 4D,E).

Overall, the results indicate that the emergence of the Laplace distribution of collective breathing intervals is robust to the choice of individual breathing interval distributions and is primarily driven by heterogeneity among individuals rather than the shape of their distributions. This is supported by the analytical theory of Sarfati et al. [1], which showed that emergent periodicity and synchrony are guaranteed for *any* input single individual distribution shape of intervals between the focus events.

Supplementary Note 3.2. Robustness to nonlinear versus linear accumulation dynamics.

To test the impact of accumulation dynamics on collective breathing interval distribution, we conducted additional simulations in which the individual dynamics in Eq. (1) were replaced with a weakly nonlinear, concave-down form:

$$\frac{dV_i}{dt} = \frac{\log\left(\frac{1}{0.01}\right)}{T_{si}}[1 - V_i(t-1)][1 - \varepsilon_i(t)] - \frac{1}{T_{di}}\varepsilon_i(t) + \sum_{j=1,j\neq i}^{N} \frac{\beta}{N}\delta_{ij}\varepsilon_j(t)[1 - \varepsilon_i(t)], \quad \textbf{(Eq. S2)}$$

where the log(1/0.01) is set such that, in the absence of social coupling, the time to the threshold (0.99) equals the experimentally observed individual breathing intervals T_{si}. Fig. S12A provides an illustrative example of linear accumulation dynamics as in Eq. (1) versus non-linear accumulation according to Eq. (S2) for two interacting individuals over time with social coupling strength $\beta = 1.3$ and refractory period $\tau_{ref} = 1.1$.

With non-linear accumulation dynamics, the mean collective breathing interval decreases with the increase of the coupling strength β, independent of the group composition (see Fig. S12B), as it was with linear accumulation rate (see Fig. 4B,G in the main text). Notably, even for smaller coupling strength, the mean breathing interval is smaller with non-linear compared to linear accumulation. The main difference is that the variability (standard deviation) of collective intervals remains rather stable with increase of social coupling strength β in case of concave-down individual accumulation rate (see Fig. S12C), whereas it increases in the linear case (see Fig. 4C,H in the main text). As a result, the match to the empirical Laplace distribution is weaker (higher KS statistics, Fig. S12D), and heterogeneous groups do not exhibit a Laplace distribution with increasing coupling strength (see Fig. S12E), unlike they do in the case of linear accumulation (see Fig. 4J,K in the main text). This way, while nonlinear, concave-down accumulation preserves the decrease of mean collective intervals with coupling, it reduces variability and weakens the emergence of Laplace-like distributions compared to linear accumulation.

Supplementary Note 3.3. Robustness to refractory period.

We tested the robustness of our agent-based model against the refractory period τ_{ref} and coupling strength β in homogenous (single breathing type) and heterogeneous (six breathing types) groups with identical coupling strength among individuals (see Fig. S5). Across both compositions, increasing β and τ_{ref} induced clear transitions in synchronization, mean collective breathing interval, and its standard deviation (Figs. S5A-C). Specifically, synchronization increased with stronger coupling and longer refractory periods in both

homogeneous and heterogeneous groups (Fig. S5A). In particular, introducing a non-zero refractory period consistently enhanced the degree of synchronization compared to $\tau_{ref} = 0$.

Figs. S5D-F show the results of the fitting of the simulated collective breathing interval distributions, generated across different combinations of β and τ_{ref}, to Laplace-like distributions. The Laplace-like distribution provides the best fit in the parameter regime beyond the transition, i.e., with stronger coupling $\beta \geq 10$ and τ_{ref} between 1.0s and 1.8s (see Fig. S5D, highlighted region). This supports our choice of $\tau_{ref} = 1.1$s for the model with homogenous coupling, placing it within the parameter regime that most closely reproduces the empirically observed synchronization and collective breathing interval statistics.
For heterogeneous coupling strength among individuals, corresponding to a cluster synchronization scenario (Fig. S8) in which only subsets of agents participated in collective breathing events, there is a transition to stronger synchronization for $\beta_{inter_{clu}} > 0.5$, which is further enhanced by longer refractory periods τ_{ref}. The sum of squared differences (SSD) between empirical and simulation-generated partaking probabilities in collective breathing events was lowest in the transition region $\beta_{inter_{clu}} \in \{0.6, 0.7\}$, and maintained robustness across different refractory periods (Fig. S8B). In contrast, the likelihood of encountering a Laplace-like distribution of collective breathing intervals, their mean and standard deviation were sensitive to changes in τ_{ref} (Fig. S8C-E). Fig. S8C highlights a specific parameter regime in which collective interval distributions are most likely to be Laplace-like. The overlap of this regime with the region of close agreement between empirical and simulated partaking probabilities (Fig. S8B) corresponds to $\beta_{inter} = 0.7$ and $\tau_{ref} \geq 1.7$s. This parameter range supports our choice of $\tau_{ref} = 2.1$s for the model with heterogenous coupling. Fig.S8F illustrates the corresponding collective breathing interval distribution and participation proportions for a simulated heterogeneous group with $\beta_{inter} = 0.7$ and $\tau_{ref} = 1.7$s, which shows no qualitative difference compared to $\tau_{ref} = 2.1$s (Fig. 6G).

Supplementary Note 3.4. Robustness of simulated group synchrony.
We also tested how an unequal distribution of breathing types affects the degree of synchrony in heterogeneous group compositions (Fig. S7). As the proportion of fast breathers increased (up to 57%) and slow breathers decreased (down to 8%), overall synchrony gradually declined from 0.99 to 0.97. Interestingly, when slow breathers were further reduced to 0%, the group regained full synchronization. Overall, the synchrony of individual breathing events in a group remained high (i.e., within the range of 0.97 to 1), suggesting robustness to group composition imbalances in the distribution of breathing types.

Supplementary Note 3.5. Impact of the fastest breathing individual type on the simulated collective breathing interval distribution.

To test the impact of the faster breathing individuals on the mean of simulated collective breathing intervals, we conducted a proof-of-concept simulations including an “imaginary” seventh individual breathing type, modeled by taking the empirically measured individual with the fastest breathing intervals (i.e., individual 6 in Fig. 2A) and subtracting 7s from all its intervals.

Fig. S9B shows that there is a parameter regime of social coupling where the collective breathing intervals follow a Laplace distribution. This regime coincides with transitions in other metrics, with the best match to the empirical data at $\beta_{intra} = 20$ and $\beta_{inter} = 1.2$. In this case, the mean of the model-generated Laplace distribution of collective breathing intervals is very close to the empirical mean ($\mu_{sim} = 14.82$ vs. $\mu_{emp} = 14.22$, see Fig. S9E). This exploratory analysis shows that very fast-breathing individuals can alter the mean of the collective breathing interval distribution, while the emergence of a Laplace distribution remains robust.

Supplementary Note 3.6. Impact of group size on simulated collective breathing.

To examine the effect of group size N on collective breathing interval distribution, we performed simulations across a broad range of N = {10, 20, 50, 100, 200, 300, 500, 800, 1000}. In all cases, groups consisted of six distinct breathing types in equal proportions, sampled from the empirical individual breathing interval distributions.

The results show that the main qualitative findings are robust to group size. Specifically, for all N, the group achieves the same mean synchrony index, with higher synchronization for stronger social coupling β (Fig. S10A). Smaller groups ($N < 100$) exhibit larger variability in collective breathing intervals (Fig. S10B,E). At weak coupling strength, the mean collective breathing interval depends strongly on group size, with larger groups ($N \geq 100$) showing longer intervals (Fig. S10C,E). As coupling strength increases, the distribution of collective breathing intervals shifts to smaller values for $N \geq 50$ (Fig. S10E). Laplace-shaped distributions emerge primarily in the parameter regime associated with a high degree of synchronization and a transition to shorter and less variable collective breathing intervals (Fig. S10D). Overall, while smaller groups exhibit larger variability in the emergent collective breathing interval, the qualitative dynamics, i.e., synchronization and shorter breathing

intervals with stronger coupling, are preserved across group sizes, consistent with the predictions of Sarfati et al. [1].

Supplementary References

1 Sarfati, R., et al., (2023) Emergent periodicity in the collective synchronous flashing of fireflies. eLife, 2023. 12: p. e78908.
