# Supplementary material for: Air-breathing synchrony in juvenile Arapaima gigas reveals collective coordination under individual physiological constraints
Source: Commun Biol. 2026 Jun 17;9:831. doi: 10.1038/s42003-026-10472-w (PMC13276053; doi:10.1038/s42003-026-10472-w)
Supplement: Supplementary file 2 — Description of Additional Supplementary Files [file 42003_2026_10472_MOESM2_ESM.pdf]

Description of Additional Supplementary Files

File name: Supplementary Data 1

Description: The source data behind the graphs in Figures 2-3 of the paper.

File name: Supplementary Video 1

Description: Recording of a shoal of Arapaima gigas (ca. 30 cm body size) in an indoor facility. Individuals swim close to each other in a cube like formation and often large parts of the shoal rapidly surface together to take new air in.

File name: Supplementary Video 2

Description: A high-speed recording (240 fps) of a collective breathing event of Arapaima gigs juveniles (ca. 30 cm body size) in an indoor facility. Before reaching the surface, fish release old air through their gills slits (bubbles visible). After fish reach the surface, a rapid cstartle behavior is initiated to return fast into deeper water.
