# Supplementary material for: Air-breathing synchrony in juvenile Arapaima gigas reveals collective coordination under individual physiological constraints
Source: Commun Biol. 2026 Jun 17;9:831. doi: 10.1038/s42003-026-10472-w (PMC13276053; doi:10.1038/s42003-026-10472-w)
Supplement: Supplementary file 6 — Reporting Summary [file 42003_2026_10472_MOESM6_ESM.pdf]

nature portfolio

Corresponding author(s): Palina Bartashevich, David Bierbach

Last updated by author(s): Apr 24, 2026

Reporting Summary

Nature Portfolio wishes to improve the reproducibility of the work that we publish. This form provides structure for consistency and transparency in reporting. For further information on Nature Portfolio policies, see our Editorial Policies and the Editorial Policy Checklist.

Statistics

For all statistical analyses, confirm that the following items are present in the figure legend, table legend, main text, or Methods section.

n/a	Confirmed	
☐	☒	The exact sample size (*n*) for each experimental group/condition, given as a discrete number and unit of measurement
☐	☒	A statement on whether measurements were taken from distinct samples or whether the same sample was measured repeatedly
☐	☒	The statistical test(s) used AND whether they are one- or two-sided<br>*Only common tests should be described solely by name; describe more complex techniques in the Methods section.*
☒	☐	A description of all covariates tested
☐	☒	A description of any assumptions or corrections, such as tests of normality and adjustment for multiple comparisons
☐	☒	A full description of the statistical parameters including central tendency (e.g. means) or other basic estimates (e.g. regression coefficient) AND variation (e.g. standard deviation) or associated estimates of uncertainty (e.g. confidence intervals)
☐	☒	For null hypothesis testing, the test statistic (e.g. *F*, *t*, *r*) with confidence intervals, effect sizes, degrees of freedom and *P* value noted<br>*Give P values as exact values whenever suitable.*
☒	☐	For Bayesian analysis, information on the choice of priors and Markov chain Monte Carlo settings
☒	☐	For hierarchical and complex designs, identification of the appropriate level for tests and full reporting of outcomes
☐	☒	Estimates of effect sizes (e.g. Cohen's *d*, Pearson's *r*), indicating how they were calculated

Our web collection on statistics for biologists contains articles on many of the points above.

Software and code

Policy information about availability of computer code

Data collection	Francisco, F. A. (2025). Recording Script for USB Basler Camera (Version 1.0.0) [Computer software]. https://github.com/fritzfrancisco/ArapaimaBehaviour; MS Excel, Basler Recording Software, Movavi Video Converter, Biotracker
Data analysis	MS Excel, SPSS 25, Prism 10, Python

For manuscripts utilizing custom algorithms or software that are central to the research but not yet described in published literature, software must be made available to editors and reviewers. We strongly encourage code deposition in a community repository (e.g. GitHub). See the Nature Portfolio guidelines for submitting code & software for further information.

Data

Policy information about availability of data

All manuscripts must include a data availability statement. This statement should provide the following information, where applicable:

- Accession codes, unique identifiers, or web links for publicly available datasets
- A description of any restrictions on data availability
- For clinical datasets or third party data, please ensure that the statement adheres to our policy

The numerical source data underlying Figs. 2-3 are provided in Supplementary Data 1. The simulation source data underlying Figs. 4-7 are available in the Zenodo repository at https://zenodo.org/records/19813940.

Research involving human participants, their data, or biological material

Policy information about studies with human participants or human data. See also policy information about sex, gender (identity/presentation), and sexual orientation and race, ethnicity and racism.

Reporting on sex and gender	NA
Reporting on race, ethnicity, or other socially relevant groupings	NA
Population characteristics	NA
Recruitment	NA
Ethics oversight	NA

Note that full information on the approval of the study protocol must also be provided in the manuscript.

Field-specific reporting

Please select the one below that is the best fit for your research. If you are not sure, read the appropriate sections before making your selection.

☐ Life sciences ☐ Behavioural & social sciences ☒ Ecological, evolutionary & environmental sciences

For a reference copy of the document with all sections, see nature.com/documents/nr-reporting-summary-flat.pdf

Ecological, evolutionary & environmental sciences study design

All studies must disclose on these points even when the disclosure is negative.

Study description	We study how individuals in large groups coordinate state-dependent behaviors shaped by internal physiological demands that may limit the ability to conform. Here, we address this question by combining agent-based modeling with empirical observations of an unprecedented case of synchronization in fish – the collective air-breathing of juvenile Arapaima gigas.
Research sample	group of ca. 200 juvenile Arapaima gigas in aquaculture facility
Sampling strategy	Video observation of individual and collective air-breathing behavior
Data collection	AEA, FAF, DB recorded the breathing events.
Timing and spatial scale	Recodings were done in Jan-Feb 2021 at Manich Food Innovations GmbH, (MFI) in Dingelstedt, Thuringia and IGB Berlin.
Data exclusions	NA
Reproducibility	We state all used tank sizes and hard- and software, animal origins and maintenance used during our observations in order to allow reproducibility
Randomization	Videos were analysed for breathing events by AEA without knowing video ID or Fish ID (in case of single individual recordings).
Blinding	NA

Did the study involve field work? ☐ Yes ☒ No

Reporting for specific materials, systems and methods

We require information from authors about some types of materials, experimental systems and methods used in many studies. Here, indicate whether each material, system or method listed is relevant to your study. If you are not sure if a list item applies to your research, read the appropriate section before selecting a response.

Materials & experimental systems

n/a	Involved in the study
☒	☐ Antibodies
☒	☐ Eukaryotic cell lines
☒	☐ Palaeontology and archaeology
☐	☒ Animals and other organisms
☒	☐ Clinical data
☒	☐ Dual use research of concern
☒	☐ Plants

Methods

n/a	Involved in the study
☒	☐ ChIP-seq
☒	☐ Flow cytometry
☒	☐ MRI-based neuroimaging

Animals and other research organisms

Policy information about studies involving animals; ARRIVE guidelines recommended for reporting animal research, and Sex and Gender in Research

Laboratory animals	Arapaima gigas
Wild animals	NA
Reporting on sex	NA
Field-collected samples	NA
Ethics oversight	Our study involved only observations of fish behavior without invasive manipulations in an aquaculture facility. No further permissions are required by german law.

Note that full information on the approval of the study protocol must also be provided in the manuscript.

Plants

Seed stocks	NA
Novel plant genotypes	NA
Authentication	NA
